# DNA Damage Signaling Is Induced in the Absence of Epstein—Barr Virus (EBV) Lytic DNA Replication and in Response to Expression of ZEBRA

**DOI:** 10.1371/journal.pone.0126088

**Published:** 2015-05-07

**Authors:** Ruth Wang'ondu, Stuart Teal, Richard Park, Lee Heston, Henri Delecluse, George Miller

**Affiliations:** 1 Department of Cell Biology, Yale University School of Medicine, New Haven, Connecticut, United States of America; 2 Department of Molecular Biophysics and Biochemistry, Yale University School of Medicine, New Haven, Connecticut, United States of America; 3 Department of Pediatrics, Yale University School of Medicine, New Haven, Connecticut, United States of America; 4 German Cancer Research Centre, Heidelberg, Germany; 5 Inserm Unit, Heidelberg, Germany; 6 Department of Epidemiology and Public Health, Yale University School of Medicine, New Haven, Connecticut, United States of America; Karolinska Institutet, SWEDEN

## Abstract

Epstein Barr virus (EBV), like other oncogenic viruses, modulates the activity of cellular DNA damage responses (DDR) during its life cycle. Our aim was to characterize the role of early lytic proteins and viral lytic DNA replication in activation of DNA damage signaling during the EBV lytic cycle. Our data challenge the prevalent hypothesis that activation of DDR pathways during the EBV lytic cycle occurs solely in response to large amounts of exogenous double stranded DNA products generated during lytic viral DNA replication. In immunofluorescence or immunoblot assays, DDR activation markers, specifically phosphorylated ATM (pATM), H2AX (γH2AX), or 53BP1 (p53BP1), were induced in the presence or absence of viral DNA amplification or replication compartments during the EBV lytic cycle. In assays with an ATM inhibitor and DNA damaging reagents in Burkitt lymphoma cell lines, γH2AX induction was necessary for optimal expression of early EBV genes, but not sufficient for lytic reactivation. Studies in lytically reactivated EBV-positive cells in which early EBV proteins, BGLF4, BGLF5, or BALF2, were not expressed showed that these proteins were not necessary for DDR activation during the EBV lytic cycle. Expression of ZEBRA, a viral protein that is necessary for EBV entry into the lytic phase, induced pATM foci and γH2AX independent of other EBV gene products. ZEBRA mutants deficient in DNA binding, Z(R183E) and Z(S186E), did not induce foci of pATM. ZEBRA co-localized with HP1β, a heterochromatin associated protein involved in DNA damage signaling. We propose a model of DDR activation during the EBV lytic cycle in which ZEBRA induces ATM kinase phosphorylation, in a DNA binding dependent manner, to modulate gene expression. ATM and H2AX phosphorylation induced prior to EBV replication may be critical for creating a microenvironment of viral and cellular gene expression that enables lytic cycle progression.

## Introduction

Infection with Epstein-Barr virus (EBV), the first tumor virus described in humans, is associated with B-cell lymphoproliferative syndromes, such as Hodgkin and endemic Burkitt lymphoma and with diseases of epithelial cell origin such as oral hairy leukoplakia, nasopharyngeal carcinoma, and gastric carcinoma [1–4]. DNA damage signaling pathways are induced during EBV infection and lytic reactivation in both lymphoid and epithelial cells [5–9]. Activation of cellular DNA damage signaling pathways, which safeguard cellular genome integrity, may indicate the presence of oncogenic stressors. Our study investigates the activation of DNA damage responses (DDR) as a consequence of EBV lytic cycle reactivation and expression of EBV lytic genes in cells of lymphoid and epithelial origin.

Phosphorylation of Ataxia telangiectasia mutated (ATM), a transducer protein in the homologous recombination (HR) pathway of DDR, is a classic marker of DNA damage signaling activation. Following initiation of DNA damage signaling due to DNA breaks or chromatin remodeling, ATM, which exists as a dimer in its inactive state, autophosphorylates at S1981 and dissociates into kinase-active monomers [10]. Upon activation, ATM phosphorylates several mediators of DNA damage signaling and repair including H2AX, a histone 2A isoform, and P53 binding protein 1 (53BP1), a scaffolding protein [10–13]. Several viral transcription activators, including HSV-1 ICP0, HIV-1 Tat protein, and HHV6 U19 protein, modulate DNA damage signaling responses and functionally interact with proteins involved in chromatin remodeling [14–17]. An emerging view is that chromatin remodeling may be a common mechanism for ATM kinase activation by viral transcription factors [18].

Reactivation of the EBV lytic cycle is characterized by a temporal cascade of viral gene expression [19]. In the very early stage of the cascade two transactivator genes, *BZLF1* and *BRLF1* encoding the ZEBRA (BamHI *Z E*pstein-*B*arr virus *r*eplication *a*ctivator) protein and Rta (R transactivator), respectively, are expressed. In subsequent stages of the lytic gene cascade, ZEBRA and Rta, independently or synergistically, activate early lytic viral genes such as *BMRF1*, which encodes EA-D, the EBV DNA polymerase processivity factor, and *BGLF4*, *BGLF5*, and *BALF2*, which encode the BGLF4 protein kinase, BGLF5 alkaline exonuclease, and BALF2 single-stranded DNA binding protein, respectively. Following expression of *BRLF1* and *BMRF1* genes, their products, Rta and EA-D, adopt distinct, lytic-phase-dependent, intranuclear localization patterns, diffuse or globular, which distinguish the early lytic phase from the late lytic cycle stage [20–22]. Diffuse intranuclear distribution of EA-D coincides with early stages of the lytic cycle during which there is no viral lytic DNA replication [21, 22]. Expression of late genes, such as *BFRF3*, which encodes the structural capsid protein FR3, is dependent on EBV lytic DNA replication.

The activity of viral proteins and introduction of viral DNA into cells are two pathways through which viral infection can trigger DNA damage signaling [23, 24]. Several early EBV proteins, Rta, BGLF4, BGLF5, and BALF2 induce phosphorylation of ATM or H2AX in EBV-negative cells [25–28]. BGLF4 has been proven to play a role in inducing ATM phosphorylation during the EBV lytic cycle in Akata cells induced into the lytic cycle by treatment with anti-IgG [26]. ATM kinase and several of its substrates are phosphorylated and recruited to viral replication compartments during activation of the EBV lytic cycle [7, 9]. These observations support the prevalent hypothesis that ATM-mediated DDR is induced in response to newly replicated viral DNA [29]. However, this hypothesis does not take into consideration the activation of ATM-mediated-DDR by early EBV proteins in the absence of viral DNA replication. Ramasubramanyan et al. (2012) showed elevated association of phosphorylated H2AX to specific regions of the EBV genome following lytic induction of Akata cells in the presence and absence of acyclovir [30]. This work provided evidence for activation of ATM-mediated DDR during the pre-replicative phase of EBV lytic induction.

We used experimental systems that allowed for isolation of early, pre-replicative, lytic events from late lytic events to identify components of the EBV lytic cycle that are associated with activation of ATM and its substrates. We show, in several cell lines, that markers of DNA damage signaling, phosphorylation of ATM (pATM) or 53BP1 (p53BP1), or H2AX (γH2AX), are activated early in the lytic cycle, in the absence of EBV lytic replication, as well as in EBV-positive cells undergoing lytic DNA replication. Upon EBV lytic reactivation, foci of pATM were induced in an epithelial cell line that did not express *BGLF4* or *BGLF5* genes. Expression of ZEBRA in EBV-negative cells induced pATM foci. Using point mutants of ZEBRA, the mechanism of ATM phosphorylation was shown to depend on ZEBRA’s capacity to bind DNA. ZEBRA colocalized with HP1β, a heterochromatin associated protein linked to ATM activation [31–33].

Our findings demonstrate a novel role for the pre-replicative stage of the EBV lytic cycle in induction of DNA damage signaling. Furthermore, our studies expand the current understanding of the role individual EBV proteins play in inducing ATM phosphorylation and provide a novel perspective on triggers of DNA damage signaling pathways during the EBV lytic cycle.

## Results

### pATM and p53BP1 foci are induced in the presence or absence of EBV DNA replication compartments

**Fig 1:**
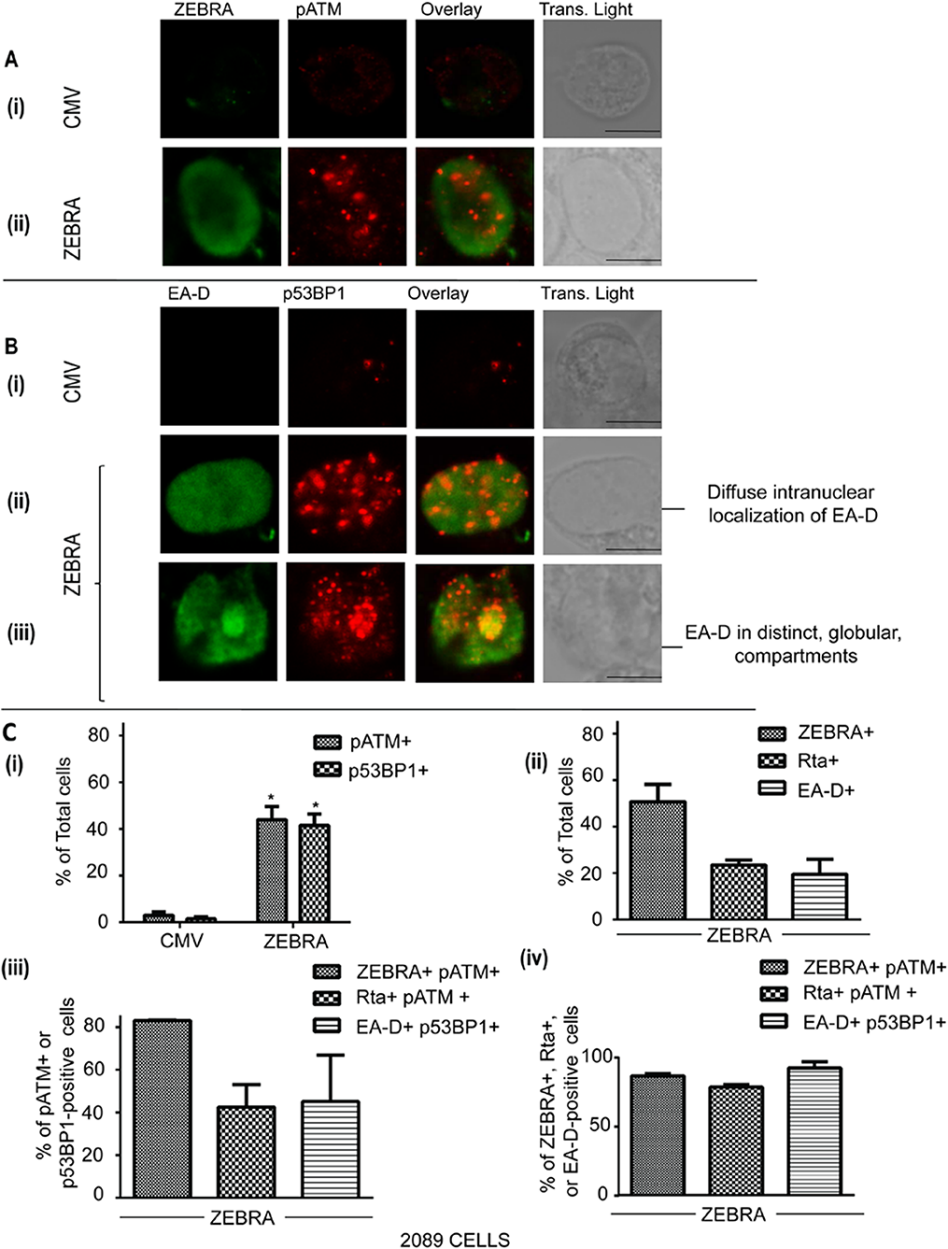
pATM and p53BP1 are induced in response to reactivation of the EBV lytic cycle. 2089 cells were transfected with an empty vector (CMV) or a plasmid bearing the wild-type ZEBRA gene. Cells were fixed and double-stained for **(A)** ZEBRA and pATM (S1981), or **(B)** EA-D and p53BP1 (S1778). EA-D-positive cells with EA-D in a diffuse **(B: ii)** or globular pattern **(B: iii)** were identified. Scale bar = 10 μm. **(C)** The percentages of cells containing DNA damage markers or lytic EBV markers were determined and the results represented as means; n = 3. The percentages of total cells that were positive for pATM or p53BP1 were determined and the results represented as average percentages. * denotes P<0.05; P = 0.047 for pATM+ cells in ZEBRA vs CMV and P = 0.048 for p53BP1+ cells in ZEBRA vs CMV **(C: i)**.The percentages of total cells that were positive for ZEBRA, Rta, EA-D were determined and the results represented as average percentages **(C: ii)**. The percentages of pATM-positive cells that were also positive for ZEBRA or Rta, and the percentages of p53BP1-positive cells that were also positive for EA-D was determined and the results represented as average percentages **(C: iii)**. The percentages of ZEBRA-positive, Rta-positive, or EA-D-positive cells that were also positive for pATM or p53BP1 were determined and the results represented as average percentages **(C: iv)**. The differences between percentages in C: ii, C: iii, and C: iv were not statistically significant (P>0.05).

**Fig 2:**
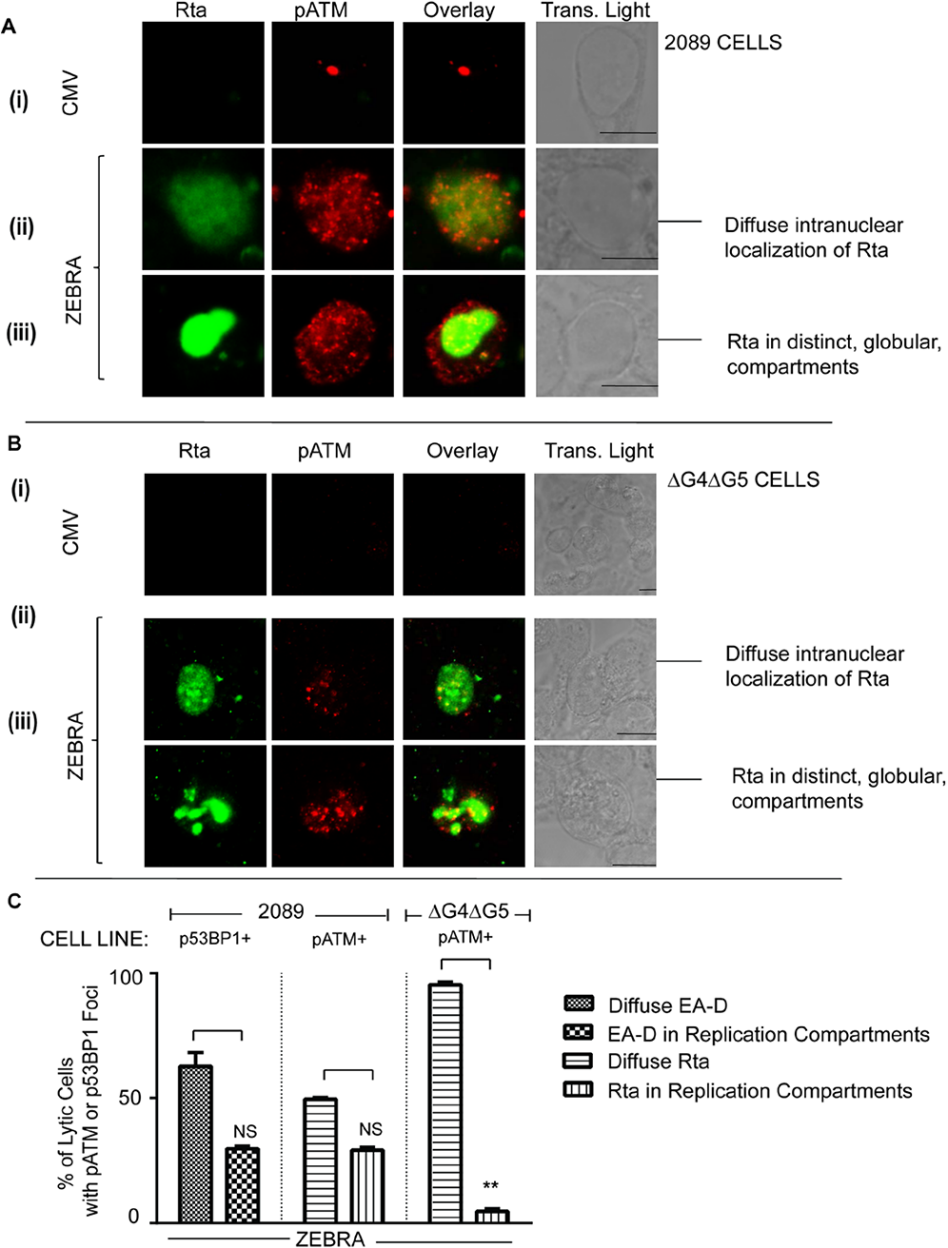
pATM and p53BP1 are induced in the presence or absence of replication compartments during EBV lytic cycle reactivation. 2089 cells **(A, C)** or ΔG4ΔG5 cells **(B, C)** were transfected with an empty vector (CMV) or a plasmid bearing the wild-type ZEBRA gene. Cells were fixed and double-stained for Rta and pATM **(A, B**). Rta-positive cells with Rta in a diffuse **(A: ii, B: ii)** or globular pattern **(A: iii, B: iii)** were identified. Scale bar = 10 μm. (**C)** The percentages of Rta and pATM-positive 2089 or ΔG4ΔG5 cells or EA-D and p53BP-positive 2089 cells where Rta or EA-D were distributed in diffuse or globular intra-nuclear patterns were determined and the results represented as average percentages **(C)** (n = 2, NS = not significant, ** denotes P = 0.00015).

During the EBV lytic cycle, EA-D and Rta proteins are localized, using immunofloresence labeling, diffusely (Fig 1B: ii, Fig 2A: ii, and Fig 2B: ii) or in globular structures (Fig 1B: iii, Fig 2A: iii and Fig 2B: iii). We studied activation of DNA damage signaling during the pre-replicative stage of the EBV lytic cycle, characterized by diffuse staining of EA-D and Rta, and during replication when these proteins are found in globular replication compartments (Fig 1 and Fig 2).

We transfected 2089 cells, carrying an inducible EBV bacmid, with an empty vector (CMV) (Fig 1A: i, Fig 1B: i, Fig 1C: i, Fig 2A: i and Fig 2B: i) or the same vector expressing ZEBRA (Fig 1A: ii, Fig 1B ii–iii, Fig 1C and 1A and Fig 2A: ii–iii, Fig 2B ii–iii and Fig 2C). Using immunofluorescence labeling, the cells were assessed, 32 hrs after transfection, for expression of ZEBRA (Fig 1A), EBV lytic proteins, EA-D (Fig 1B) or Rta (Fig 2A), and markers of DNA damage signaling, namely pATM (Figs 1A, 1C and 2A) and p53BP1 (Fig 1B and 1C). Intranuclear foci of pATM (Fig 1A: ii and Fig 2A ii–iii) and p53BP1 (Fig 1C: i) were induced upon expression of ZEBRA in 2089 cells. A significantly greater percentage of cells transfected with the ZEBRA expression vector contained foci of pATM (40–48%) or p53BP1 (38–45%) compared to cells transfected with an empty vector (2–4% pATM-positive and 1–2% p53BP1-positive cells; Fig 2C: i). ZEBRA was expressed in 46–56% (Fig 1A: ii and Fig 1C: ii), EA-D in 15–24% (Fig 1B: ii–iii and Fig 1C: ii), and Rta in 22–25% (Fig 2A: ii–iii and Fig 1C: ii), of cells transfected with the ZEBRA expression vector.

pATM foci were induced in cells expressing ZEBRA (Fig 1: ii) as well as in lytically reactivated cells expressing Rta (Fig 2A: ii–iii). Foci of p53BP1, a substrate of pATM, were expressed in cells expressing EA-D (Fig 1B: ii–iii). A majority of cells containing foci of pATM (83%) were also positive for ZEBRA (Fig 1A: ii and Fig 1C: iii). Depending on the experiment, 35–50% of pATM-positive cells were Rta-positive (Fig 2A ii–iii, Fig 1C: iii) and 30–60% of p53BP1-positive cells were EA-D-positive (Fig 1B: ii–iii and Fig 1C: iii). Foci of p53BP1 and pATM were induced in the majority of ZEBRA-positive and lytic cells. 86–88% of ZEBRA-positive cells contained pATM foci, 90–96% of EA-D-positive cells contained p53BP1 foci and 77–80% of Rta-positive cells contained pATM foci (Fig 1C: iv). These data showed that two classical markers of DNA damage signaling are activated in response to expression of ZEBRA in the vast majority of lytically-reactivated 2089 cells.

Foci of p53BP1 and pATM were induced in cells expressing Rta or EA-D, irrespective of the intranuclear localization pattern of these EBV lytic antigens. In p53BP1-positive cells where EA-D was localized exclusively in a diffuse pattern (59–67% of cells expressing EA-D), p53BP1 foci were spread evenly over the entire nucleus (Figs 1B: ii and 2C). In 29–31% of EA-D-positive cells, EA-D and p53BP1 accumulated in globular structures within the nucleus (Figs 1B: ii and 2C). Fifty percent of Rta-positive cells contained diffusely distributed Rta and pATM foci (Fig 2A: ii and Fig 2C). In 28–30% of Rta- positive cells, both Rta and pATM localized to distinct globular viral replication compartments (Fig 2A: iii and Fig 2C). These immunofluorescence microscopy data suggest that activation of DNA damage signaling markers, pATM and p53BP1, during the EBV lytic cycle is not restricted to cells undergoing EBV DNA replication, as indicated by viral replication compartments.

### ATM is phosphorylated upon reactivation of the EBV lytic cycle in the absence of the *BGLF4* and *BGLF5* early lytic gene products

The induction of pATM foci in lytically-reactivated 2089 cells in which EA-D and Rta were diffusely distributed and not localized to globular intranuclear structures suggested that DNA damage signaling occurs in the absence of EBV DNA replication. We hypothesized that EBV proteins expressed early in the EBV lytic cycle, prior to DNA synthesis, may mediate pATM activation in the lytic pre-replicative phase. Two early lytic proteins, BGLF4, a kinase, and BGLF5, a nuclease, can induce DNA damage responses [26–28, 34].

**Fig 3:**
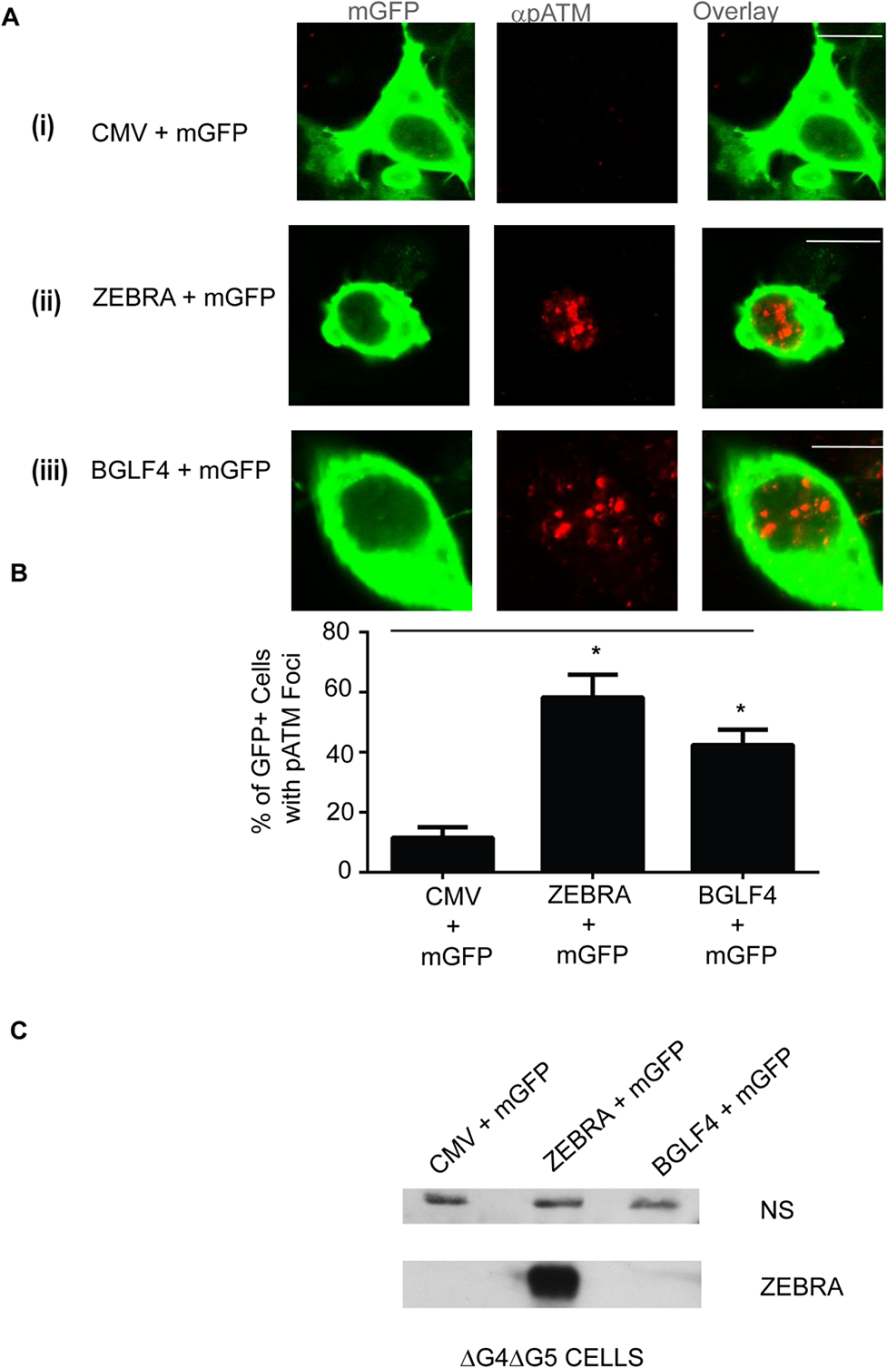
pATM is induced in response to expression of BGLF4 in ΔG4ΔG5 cells. **(A)** ΔG4ΔG5 cells were transfected with an empty vector (CMV); **(A: i)**, or a plasmid bearing the wild-type *ZEBRA* gene **(A: ii)**, or a plasmid bearing the wild-type *BGLF4* gene **(A: iii)**, together with a plasmid bearing a membrane targeted GFP gene (mGFP) then fixed and stained for pATM (S1981). Scale bar = 10 μm. **(B)** The percentages of transfected cells (based on mGFP staining) that also contained pATM foci were determined and the results represented as averages; n = 2. * denotes P<0.05; P = 0.016 for CMV+mGFP versus ZEBRA+mGFP and P = 0.019 for CMV+mGFP versus BGLF4+mGFP. **(C)** Cell lysates were analyzed by immunoblots with antibodies against ZEBRA. A non-specific band (NS) is shown as a loading control.

To determine whether BGLF4 and BGLF5 are required for ATM phosphorylation during the EBV lytic cycle, foci of pATM and expression of Rta were simultaneously assessed in ΔG4ΔG5 cells, which do not express *BGLF4* or *BGLF5* genes. ΔG4ΔG5 cells were co-transfected with plasmids bearing the genes encoding mGFP, as a marker for transfected cells, and ZEBRA proteins. Foci of pATM were induced in a significant proportion (53–64%) of mGFP-positive cells that were co-transfected with a ZEBRA expression vector compared to empty vector controls (9–14%; Fig 3).

BGLF4 and BGLF5 proteins are expressed as early lytic genes but are important for late gene expression and replication [26, 35]. In our experiments the absence of BGLF4 and BGLF5 reduced formation of replication compartments. In ΔG4ΔG5 cells, Rta was diffusely distributed in a significantly greater proportion of cells (95–96%) and only present in replication compartments in a minor fraction (4–5%) of Rta-positive cells (Fig 2B: ii and 2C). By contrast, in 2089 cells, Rta was diffusely distributed or localized to replication compartments in approximately equal proportions of Rta-positive cells (Figs 1B, 2A and 2C). Therefore, in the absence of BGLF4 and BGLF5, the population of lytic cells was enriched in the pre-replicative stage. Foci of pATM were present in the vast majority of Rta-positive ΔG4ΔG5 cells where the Rta protein was diffusely distributed in the nucleus (Fig 2B: ii), and in the few cells in which Rta was recruited to viral replication compartments (Fig 2B: iii). These data indicate that BGLF4 and BGLF5 are dispensable for the pre-replication phase induction of pATM.

We also tested the possibility that BGLF4 might support induction of ATM phosphorylation in ΔG4ΔG5 cells. pATM foci were induced in a significantly greater proportion of cells co-transfected with *BGLF4* and mGFP genes (42%–48% of mGFP-positive cells) compared to cells co-transfected with empty vector and the mGFP gene (17–19% of mGFP-positive cells) (Fig 3A and 3B). The lytic cycle, indicated by Rta expression, was activated upon transfection of ΔG4ΔG5 cells with the gene encoding ZEBRA (Fig 2B). The proportion of pATM-positive cells was similar in ΔG4ΔG5 cells transfected with *BZLF1* and mGFP genes (60–68% of mGFP-positive cells) as in cells transfected with *BGLF4* and mGFP genes (42%–48% of mGFP-positive cells). ZEBRA was not expressed in cells transfected with *BGLF4* and mGFP genes (Fig 3C). These data show that ATM is phosphorylated in response to BGLF4 expression in ΔG4ΔG5 cells, in the absence of EBV lytic reactivation, as reported in other cell types [26, 27]. Therefore, although *BGLF4* expression can generate pATM foci in the absence of EBV reactivation, it is dispensable for ATM phosphorylation in lytically reactivated EBV-positive ΔG4ΔG5 cells.

### Induction of γH2AX during the EBV lytic cycle in Burkitt lymphoma cell lines is independent of viral DNA replication

The presence of pATM foci in lytically-reactivated 2089 cells with diffuse intranuclear distribution EA-D and Rta showed that DNA damage signaling can occur in the absence of replication compartments, when the EBV lytic cycle is induced by ZEBRA expression (Figs 1 and 2). We studied Burkitt lymphoma (BL) cell lines to determine whether DNA damage signaling can occur in the absence of lytic EBV DNA replication in lymphoid cells induced into the lytic cycle by various stimuli.

**Fig 4:**
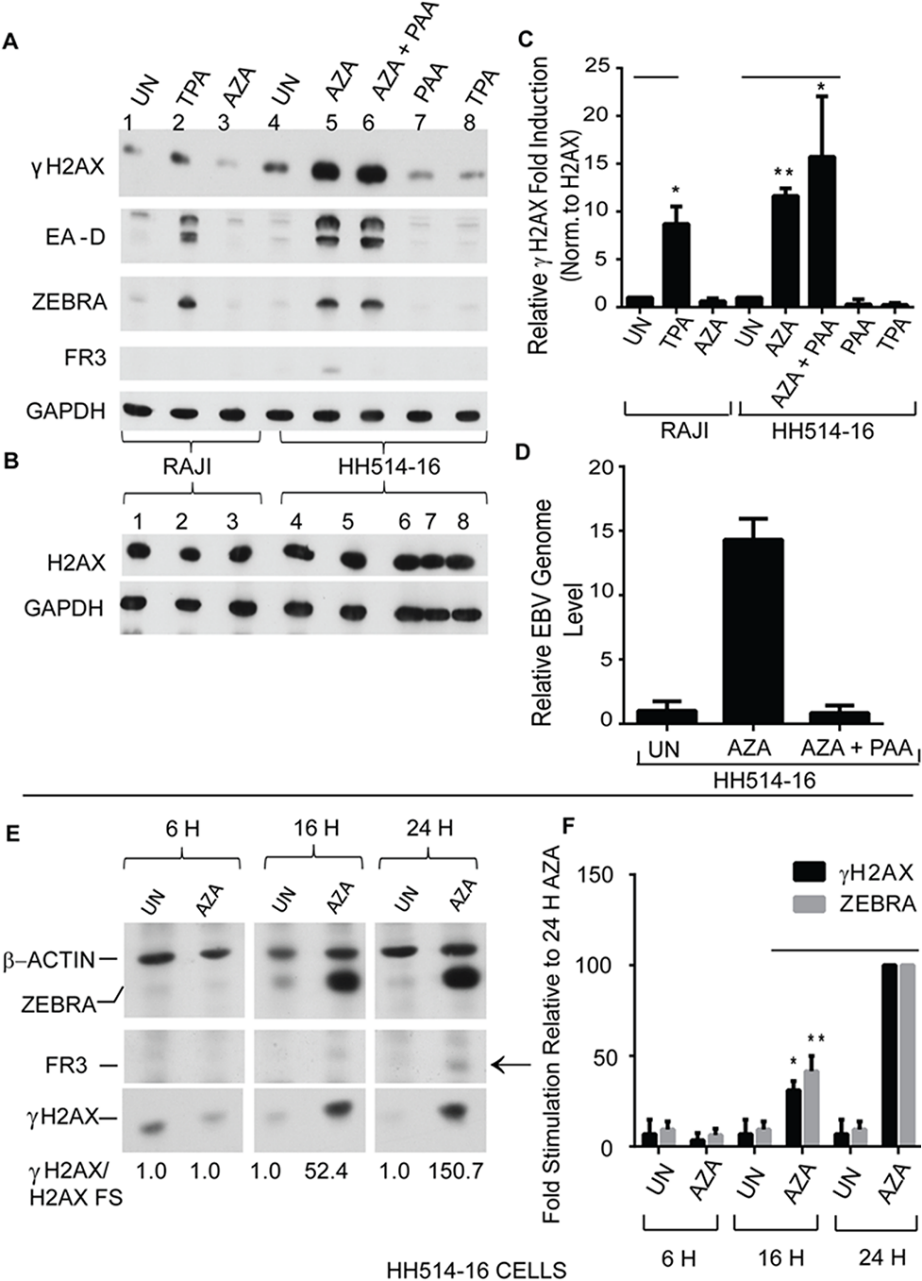
γH2AX is induced upon EBV lytic reactivation in Burkitt lymphoma cells in the presence or absence of viral DNA replication and coincides with expression of ZEBRA. **(A and B)** Raji and HH514-16 cells were not treated (UN) or treated with phorbol-12-myristate-13-acetate (TPA) or 5-aza-2’-deoxycytidine (AZA); HH514-16 cells were also treated with AZA and phosphonoacetic acid (AZA + PAA) or PAA alone for 24 hours. Cell lysates were analyzed by immunoblots with antibodies against: **(A)** γH2AX (S135), EA-D, ZEBRA, FR3, and GAPDH or **(B)** GAPDH and H2AX. **(C)** The average fold-induction of γH2AX normalized to non-phosphorylated H2AX was determined (n = 3). * denotes P<0.05; ** denotes P<0.01; P = 0.027 for TPA versus UN in RAJI cells. P = 0.00013 for AZA versus UN and P = 0.022 for AZA+PAA versus UN in HH514-16 cells. **(D)** The relative EBV genome level for the experimental samples in Fig 4A lane 4, 5, and 6 was determined by real time PCR with primers specific for the EBV brlf1 promoter. The data shown represent the average of 3 technical replicates. **(E)** Cell lysates from HH514-16 cells not treated (UN) or treated with 5-aza-2’-deoxycytidine (AZA) for 6, 16, or 24 hours were analyzed by immunoblots with antibodies against γH2AX (S135), β-actin, ZEBRA, and FR3; the average fold-induction values of γH2AX based on densitometry values of γH2AX bands normalized to unphosphorylated H2AX bands are indicated. **(F)** The average fold-induction values for γH2AX normalized to β-actin in untreated cells, relative to γH2AX induction in the cells treated with AZA for 24 hours was determined (n = 2). *denotes P<0.05; ** denotes P<0.01; P = 0.010 for ZEBRA in AZA-16 H versus in AZA-24H and P = 0.0026 for γH2AX in AZA-16 H versus in AZA-24H.

Twenty four hours after treatment of HH514-16 BL cells with AZA, ZEBRA, EA-D, and FR3, a late protein (Fig 4A, lane 5), were expressed and EBV genome amplification was detected (Fig 4D). Greater than 10-fold induction of γH2AX was detected in AZA-treated HH514-16 cells expressing ZEBRA and FR3, while the unphosphorylated form, H2AX, remained unchanged (Fig 4B, lane 5). While treatment of HH514-16 cells with AZA activated the EBV lytic cycle and induced γH2AX (Fig 4A, lane 5), treatment of cells from the Raji EBV-positive BL cell line with AZA did not induce the EBV lytic cycle or activate expression of γH2AX (Fig 4A, lane 3). These data show that activation of DNA damage signaling markers in AZA-treated HH514-16 cells correlates with induction of the EBV lytic cycle and is not likely to be an effect of AZA itself.

To determine whether induction of γH2AX during the EBV lytic cycle was strictly dependent on viral DNA replication, levels of γH2AX were measured in HH514-16 cells treated with AZA with or without addition of phosphonoacetic acid (PAA), a specific inhibitor of the EBV viral DNA polymerase [36]. After 24-hour treatment of HH514-16 cells with AZA and PAA, lytic EBV DNA was at background levels (Fig 4D). The early lytic proteins EA-D and ZEBRA were detected in HH514-16 cells treated with AZA or AZA and PAA (Fig 4A; lanes 5 and 6). FR3, a late protein whose expression is dependent on lytic DNA replication, was not expressed in cells treated with AZA and PAA (Fig 4A, lane 6; Fig 4C). γH2AX was induced to an equal level (10-fold) in HH514-16 cells treated with AZA, where lytic EBV DNA was actively replicating, as in cells treated with AZA and PAA, where viral DNA synthesis was inhibited (Fig 4A, lanes 5–6 and Fig 4C). In a time-course experiment, γH2AX induction coincided with lytic reactivation and expression of ZEBRA, while levels of H2AX did not change markedly (S1 Fig). γH2AX was induced after 16 h and 24 h of treatment of HH514-16 cells with AZA, when ZEBRA protein was expressed, but not 6h after treatment with AZA, when ZEBRA protein was not detected (Fig 4E–4F). FR3, a capsid protein expressed during the late phase of the lytic cycle after EBV replication has occurred, was only detected 24 hours after treatment with AZA. These data showed that γH2AX can be induced prior to late stages of the EBV lytic cycle and when lytic viral DNA replication is inhibited.

**Fig 5:**
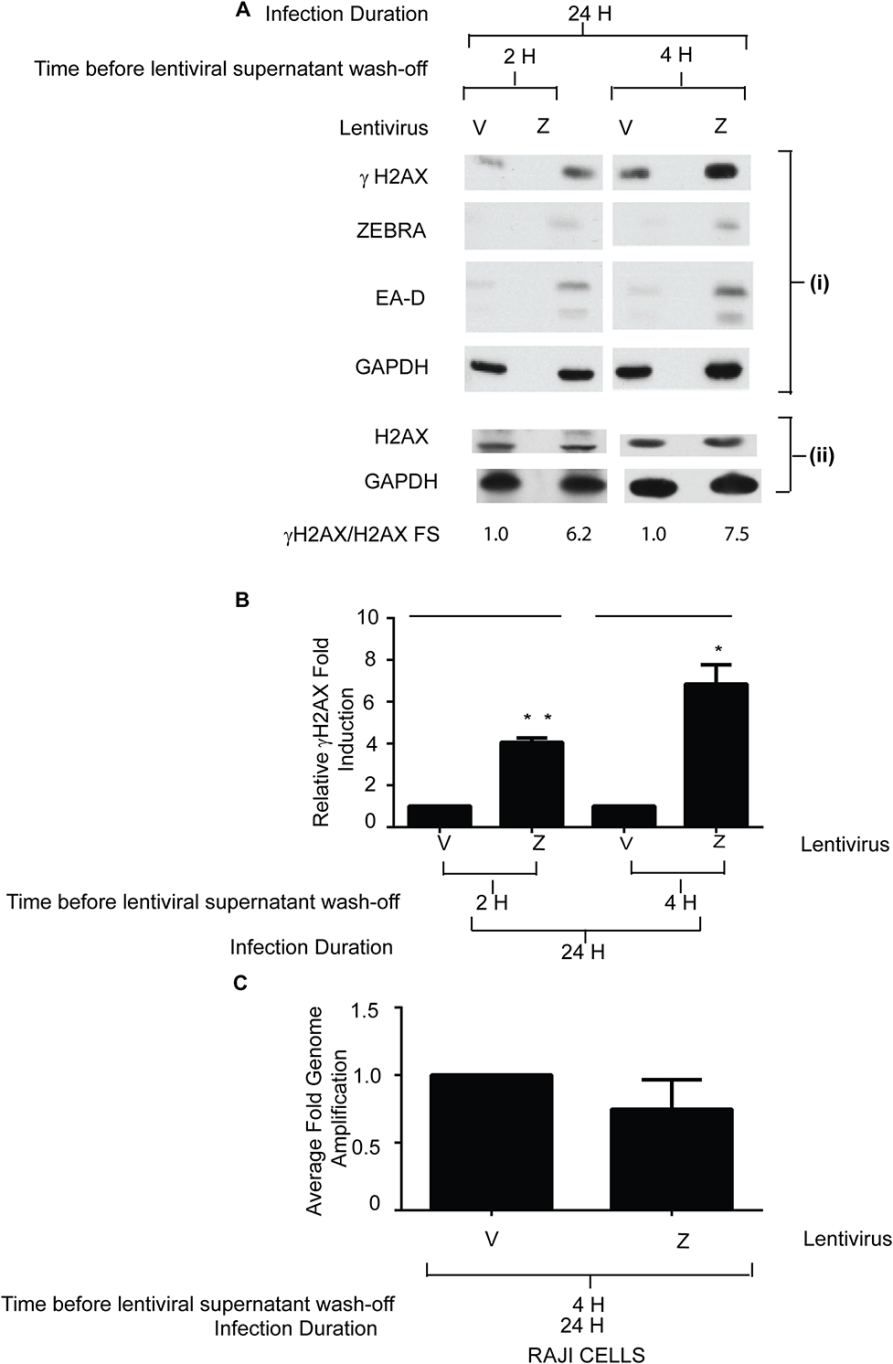
γH2AX is activated in Raji cells induced into the lytic cycle by expression of ZEBRA. Raji cells were infected with an empty vector lentivirus (V) or a lentivirus bearing the *bzlf1* gene (Z) for 2h or 4h. After infection, cells were washed and incubated for an additional 22 or 20 hours, for a total of 24 hours. **(A)** Immunoblots with antibodies against ZEBRA, γH2AX (S135), EA-D, and GAPDH **(A: i)** and H2AX and GAPDH **(A: ii)** are shown. **(B)** The average fold-induction of γH2AX by ZEBRA relative to vector transfected cells, normalized to GAPDH was determined (n = 2). * denotes P<0.05; ** denotes P<0.01; P = 0.0024 for Z-2H versus V-2H and P = 0.012 for Z-4H versus V-4H. **(C)** EBV genome amplification for Raji cells infected with an empty vector lentivirus (V) or a lentivirus bearing the ZEBRA gene (Z) for 2h was determined by real time PCR with primers specific for the EBV *brlf1* promoter. The data shown represent the average of 3 technical replicates.

To confirm that γH2AX is induced in the early stage of the EBV lytic cycle, in the absence of viral DNA replication, we used the Raji BL cell line. Raji cells cannot replicate EB viral DNA due to a deletion of the viral *BALF2* gene, encoding an indispensable replication protein [37]. ZEBRA and EA-D were induced Raji cells treated with phorbol-12-myristate-13-acetate (TPA) (Fig 4A, lane 2); FR3 late protein was not detected (Fig 4A, lane 2). Approximately 8-fold induction of γH2AX was detected in lytically active TPA-treated Raji cells (Fig 4A, lane 2, and 4C). Unlike Raji cells, HH514-16 cells are not lytically induced by TPA. To determine whether TPA induces γH2AX independent of its effect on EBV reactivation, HH514-16 cells were treated with TPA. γH2AX was not detected in HH514-16 cells treated with TPA alone (Fig 4A, lane 8). Lytic reactivation of Raji cells by transduction with a lentivirus bearing the *BZLF1* gene resulted in γH2AX activation (Fig 5A and 5B). EBV genome amplification in Raji cells expressing ZEBRA after lentivirus transduction was indistinguishable from the negative control (Fig 5C). These data showed that DDR signaling was not unique to chemical reactivation by TPA in Raji cells. Moreover, the experiments in Raji cells emphasize the conclusion that DDR signaling does occur in the absence of lytic EBV replication. Furthermore, BALF2, which has been shown to induce γH2AX in a proportion of EBV-negative nasopharyngeal cancer cells [25], and is not present in Raji cells, was not necessary for early lytic phase.

### Foci of phosphorylated ATM are induced exclusively in lytic cells, independent of viral DNA replication, during EBV reactivation in Burkitt lymphoma cell lines

**Fig 6:**
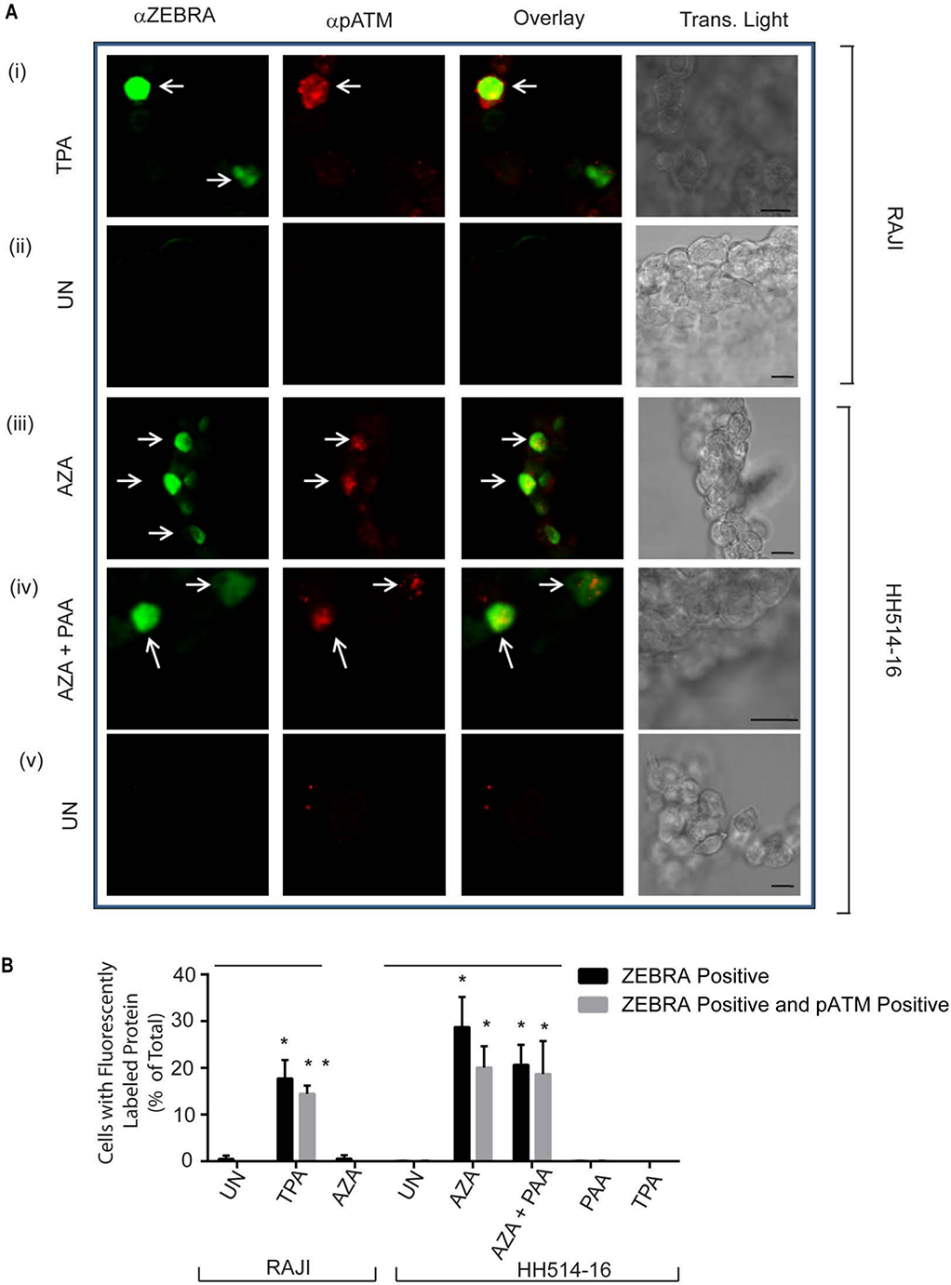
pATM is induced exclusively in lytic cells following chemical induction of the EBV lytic cycle in Burkitt lymphoma cell lines. **(A)** Raji cells treated with TPA **(A: i)** or left untreated (UN) **(A: ii)** and HH514-16 cells treated with AZA **(A: iii)**, or with AZA and PAA **(A: iv)**, or left untreated (UN) **(A: v)** were double-stained for ZEBRA and pATM (S1981). **(B)** The percentages of total cells that were positive for both ZEBRA and pATM were obtained and the results represented as averages (n = 3). * denotes P<0.05 and ** denotes P<0.01; for the average percentages of ZEBRA+ cells P = 0.027 in TPA versus UN RAJI samples and P = 0.025 for AZA versus UN HH514-16 samples. For the average percentages of ZEBRA+ and pATM+ cells P = 0.021in TPA versus UN RAJI samples, P = 0.0070 in AZA versus UN, and P = 0.047 in AZA+PAA versus UN HH514-16 samples. Scale bar = 10 μm.

We investigated whether pATM foci were induced during EBV reactivation in HH514-16 or Raji cells in the same experimental samples in which induction of γH2AX was demonstrated (Fig 4A–4D). Foci of pATM were present exclusively in TPA-treated Raji cells (Fig 6A: i), in AZA-treated (Fig 6A: iii), and AZA-and-PAA-treated (Fig 6A: iv) HH514-16 cells expressing ZEBRA, and not in similarly treated cells where ZEBRA was not present. Therefore, in both HH514-16 and Raji cells, pATM foci were only detected in cells where EBV was reactivated (Fig 6). Foci of pATM were detected in a significant proportion (approximately 20%) of HH514-16 cells activated into the lytic cycle by AZA treatment, relative to untreated cells (Fig 6: iii). A similar percentage (18–20%) of AZA-treated and AZA-and-PAA-treated HH514-16 cells were positive for both ZEBRA and pATM (Fig 6B). Approximately 15% of TPA-treated Raji cells were positive for both ZEBRA and pATM. These data showed that ATM was phosphorylated in lytically-reactivated HH514-16 cells in which lytic EBV DNA replication was chemically inhibited with PAA, as well as in lytically-reactivated, replication-deficient, Raji cells. Treatment of Raji cells with AZA or HH514-16 cells with TPA (S3 Fig), stimuli that did not induce the EBV lytic cycle in these cell lines, did not induce pATM foci. Therefore, ATM phosphorylation was induced specifically in response to induction of the EBV lytic cycle and not as a general response to treatment with chemical reagents used to induce EBV. Furthermore, these data show that BALF2, which has been shown to induce markers of DNA damage signaling [25], was not necessary for formation of pATM foci in lytically activated Raji cells.

### Induction of γH2AX is not sufficient for activation of the EBV lytic cycle

Phosphorylation of ATM during the EBV lytic cycle in HH514-16 and Raji cells (Figs 4–6) raised the question whether ATM-dependent DNA damage signaling, as shown by induction of γH2AX, is sufficient for activation of the EBV lytic cycle in these cell lines. To investigate this question, we studied lytic reactivation in HH514-16 or Raji cells treated with reagents, including sodium butyrate (NaB), ionizing radiation (IR), or camptothecin, which induce ATM kinase activity and subsequent phosphorylation of H2AX at serine 135 (γH2AX) [10, 38–44].

**Fig 7:**
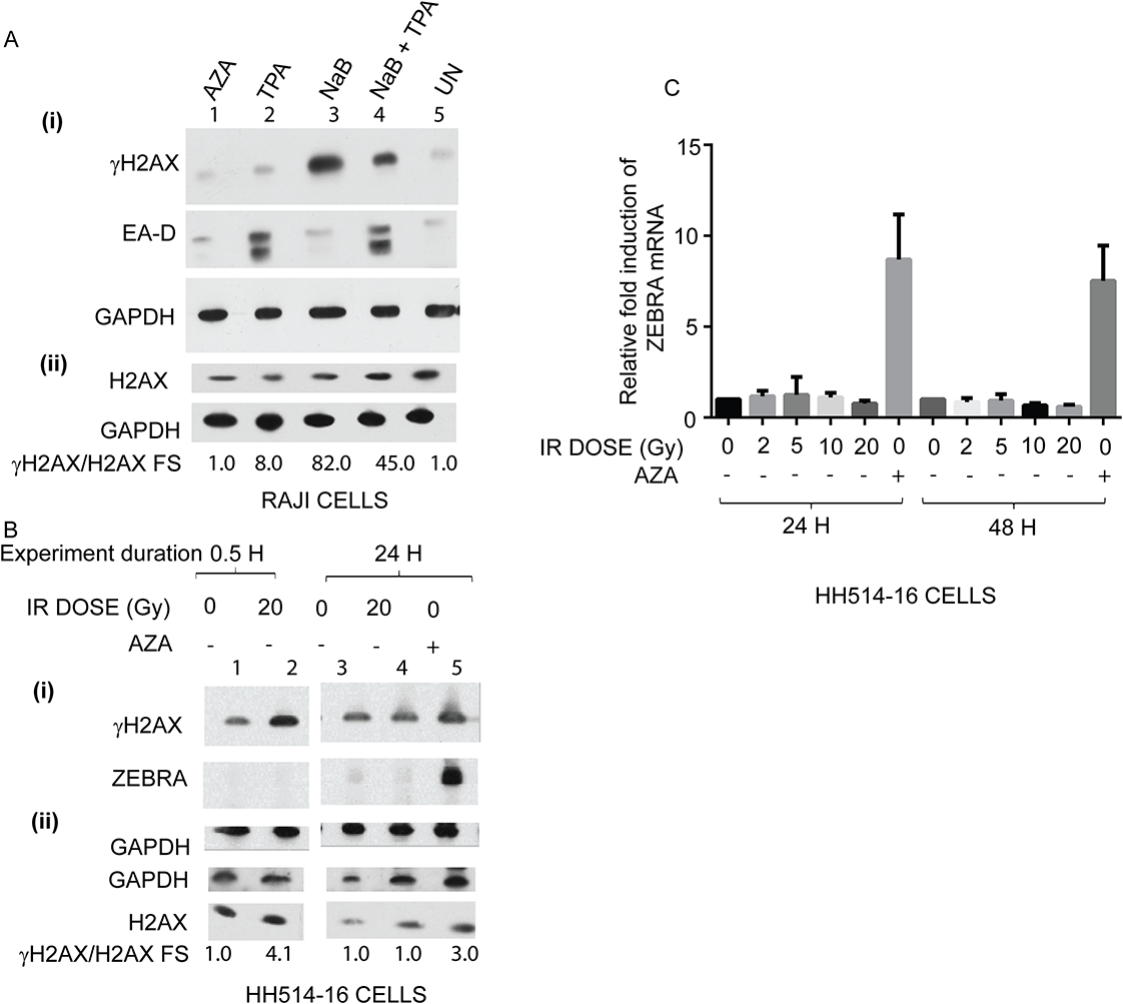
Induction of γH2AX is not sufficient for activation of the EBV lytic cycle in Burkitt lymphoma cell lines. **(A)** Cell lysates of Raji cells treated with AZA, TPA, sodium butyrate (NaB), NaB and TPA, or untreated (UN) were analyzed by immunoblots with antibodies against γH2AX, EA-D, and GAPDH **(A: i)** or H2AX and GAPDH **(A: ii)**; fold-stimulation values of γH2AX normalized to H2AX are indicated. **(B)** HH514-16 cells were irradiated with 0 Gy or 20 Gy dose of ionizing radiation (IR) and harvested after 0.5 h or 24 h, or treated with AZA for 24 h. Cell lysates were analyzed by immunoblots with antibodies against γH2AX (S135), ZEBRA, and GAPDH **(B: i)** or GAPDH and H2AX **(B: ii);** fold-stimulation values of γH2AX normalized to H2AX are indicated. **(C)** HH514-16 cells were irradiated with 0 Gy, 2Gy, 5 Gy, 10 Gy, 20 Gy, or treated with AZA. Total RNA was extracted from cell lysates after 24 h or 48 h and relative levels of BZLF1 mRNAs were measured by qRT-PCR using the standard-curve method. Individual samples were assayed in triplicate; in two biological replicates.

We tested the effects of NaB, a histone deacetylase inhibitor (HDACi), on lytic reactivation and γH2AX induction in Raji cells. Treatment of Raji cells with NaB resulted in robust (approximately 80-fold) induction of γH2AX but did not activate the EBV lytic cycle, as shown by the absence of EA-D protein (Fig 7A, lane 3). As previously illustrated (Fig 4), treatment of Raji cells with TPA induced expression of ZEBRA (Fig 7A, lane 2). Induction of γH2AX in TPA-treated Raji cells (Fig 7A, lane 2) was modest (8-fold) compared to NaB-treated cells (Fig 7A, lane 3). Treatment of Raji cells with both NaB and TPA led to a 6-fold increase of the level of γH2AX relative to Raji cells treated with TPA alone. However, levels of EA-D expression were similar in both samples (Fig 7A, compare lane 4 with lane 2). There was approximately 50% reduction in γH2AX induction in NaB-and-TPA-treated Raji cells relative to NaB-treated cells (Fig 7A, compare lane 4 with lane 3), but no corresponding decrease in the level of EA-D. These experiments in Raji cells showed no correlation between the extent of expression of γH2AX, a marker of DNA damage signaling, and EA-D, a marker of EBV lytic cycle activation.

**Fig 8:**
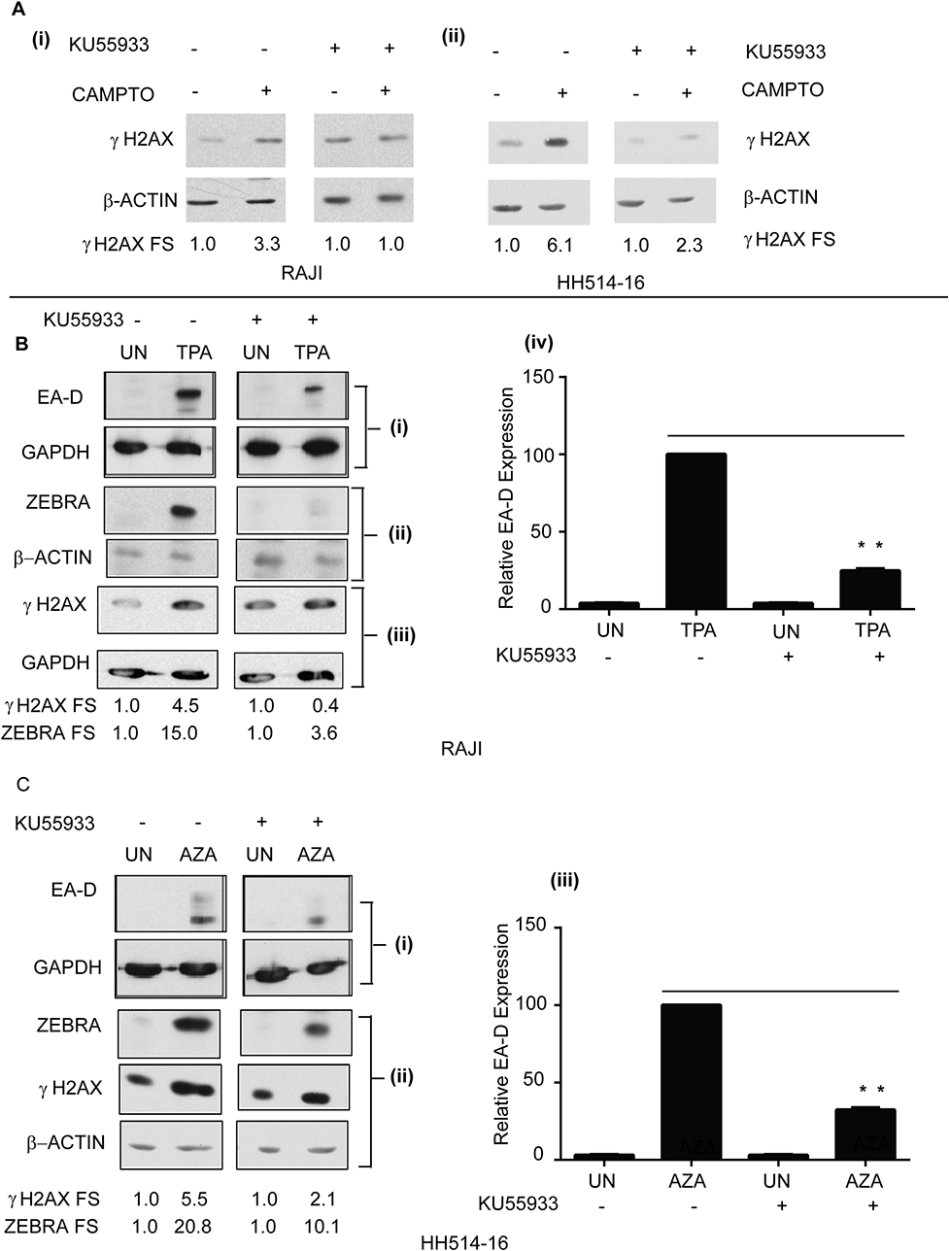
ATM kinase activity mediates maximal reactivation of the EBV lytic cycle in Burkitt lymphoma cells. **(A)** Cell lysates from Raji **(A: i)** and HH514-16 cells **(A: ii)** untreated or treated with camptothecin (CAMPTO), in the presence or absence of KU55933, were analyzed by immunoblots with antibodies against γH2AX (S135) and GAPDH; fold induction of γH2AX normalized to unphosphorylated H2AX are shown. **(B)** Cell lysates of Raji cells untreated, or treated with TPA, in the presence or absence of KU55933, were analyzed by immunoblots with antibodies against EA-D and GAPDH **(B: i)**, ZEBRA and β-Actin **(B: ii)**, and γH2AX (S135) and GAPDH **(B: iii)**; The fold stimulation values of EA-D and ZEBRA normalized to GAPDH or γH2AX normalized to unphosphorylated H2AX are shown. The average fold-induction of EA-D normalized to GAPDH was determined (n = 3); ** denotes P<0.01; P = 0.00013 for TPA+KU55933 versus TPA **(B: iv). (C)** Cell lysates of HH514-16 cells untreated, or treated with AZA, in the presence or absence of KU55933, were analyzed by immunoblots with antibodies against EA-D and GAPDH **(C: i)** and ZEBRA, γH2AX, and β-Actin **(C: ii)**; The fold stimulation values of EA-D, ZEBRA, and γH2AX are shown. The average fold-induction of EA-D normalized to GAPDH was determined (n = 3). ** denotes P<0.01; P = 0.00020 for AZA+KU55933 versus AZA **(C: iii)**.

There was a 4-fold induction of γH2AX after 30 min of treatment of HH514-16 cells with a 20 Gy dose of IR (Fig 7B, lane 2). As expected from the known kinetics of γH2AX induction [45], levels of γH2AX in irradiated cells returned to baseline 24 h after exposure to a 20 Gy dose of IR (Fig 7B, lane 3 and 4 to lane 1 and 2). ZEBRA protein was induced in response to AZA treatment but was absent 30 min and 24 h after exposure to a 20 Gy dose of IR (Fig 7B; compare lane 5 with lane 4 and 2). Transcription of the *BZLF1* gene was not activated 24 h or 48 h after treatment with increasing doses of IR, delivered at 1.46 Gy/min (Fig 7C). In experiments with camptothecin, there was a 6-fold induction of γH2AX in HH514-16 cells treated with camptothecin for 2 hours (Fig 8A: ii) and a 3-fold induction of γH2AX in Raji cells after treatment with camptothecin for 2 hours (Fig 8A: i). ZEBRA protein was not detected 22 hours after treatment of HH514-16 cells (S2A Fig) or Raji cells (S2B Fig) with camptothecin for 2 hours. These data indicate that induction of γH2AX by canonical DNA damaging reagents is not sufficient to reactivate the EBV lytic cycle in Raji or HH514-16 cells.

### Treatment of HH514-16 and Raji Burkitt Lymphoma cell lines with an ATM inhibitor prevents maximal ZEBRA and EA-D expression

To investigate whether ATM kinase activity is necessary for induction of the EBV lytic cycle in HH514-16 and Raji we studied EBV reactivation in the presence of an ATM kinase inhibitor, KU55933 [46]. To verify that the inhibitor was functional we assayed γH2AX induction in the presence or absence of KU55933. After 2h of treatment of Raji cells with camptothecin and KU55933, induction of γH2AX was inhibited by approximately 70% compared to camptothecin-treated Raji cells (Fig 8A, i). Compared to Raji cells treated with TPA, treatment of Raji cells with TPA and KU55933 resulted in a 90% reduction of γH2AX activation (Fig 8B, iii), and approximately 75% inhibition of both EA-D (Fig 8B, i and iv) and ZEBRA (Fig 8B, ii) protein expression. There was 60% inhibition of γH2AX induction after treatment of HH514-16 cells with KU55933 and camptothecin for 2h relative to cells treated with camptothecin alone (Fig 8A, ii). Compared to HH514-16 cells treated with AZA, treatment of HH514-16 cells with AZA and KU55933 resulted in a 90% reduction in γH2AX activation (Fig 8C, ii), approximately 50% inhibition of ZEBRA (Fig 8C, ii), and 70% inhibition of EA-D proteins (Fig 8C, i and iii). These data suggest that ATM kinase activity is required for maximal activation of the EBV lytic cycle.

### ZEBRA is sufficient to induce ATM phosphorylation and γH2AX in EBV-negative 293 cells

Our data in BL cells showed that DNA damage signaling occurs early during the EBV lytic cycle and is not dependent on viral lytic replication. Three EBV proteins expressed during the early lytic cycle, BGLF4, BGLF5, and BALF2 induce markers of DNA damage signaling, including γH2AX, in EBV-negative cells [25, 26, 34]. In our experiments, we showed that BGLF4 and BGLF5 proteins were not essential for early lytic phase induction of pATM foci (Figs 2B and 3B). The detection of pATM foci or γH2AX in the absence of BGLF4, BGLF5, and BALF2 in lytically-reactivated ΔG4ΔG5 and Raji cell lines led us to conclude that these proteins may not be the only EBV proteins that contribute to induction of markers of DNA damage signaling in the pre-replicative stage of the EBV lytic cycle (Figs 3–5). Our subsequent studies explored the hypothesis that ZEBRA may activate DNA damage signaling during the EBV lytic cycle.

ZEBRA is sufficient and indispensable for EBV lytic reactivation and is expressed during the very early phase of the lytic cycle [47]. Two observations in our previous experiments led us to hypothesize that ZEBRA may play a direct role in activating markers of DNA damage signaling. First, in experiments in 2089 cells, where the EBV lytic cycle was induced by transfection with a ZEBRA expression vector, the percentage of pATM-positive cells containing ZEBRA was greater than the percentage of pATM-positive cells containing Rta (Fig 1C: iii). This finding could be explained if expression of ZEBRA was sufficient to activate pATM in some cells, without activating downstream lytic genes. Secondly, induction of γH2AX in HH514-16 cells temporally correlated with expression of ZEBRA (Fig 4E). To determine whether ZEBRA is sufficient for induction of markers of DNA damage signaling, we expressed ZEBRA in 293 cells, the parental cell line for the EBV-bacmid bearing 2089 and ΔG4 ΔG5 cells.

**Fig 9:**
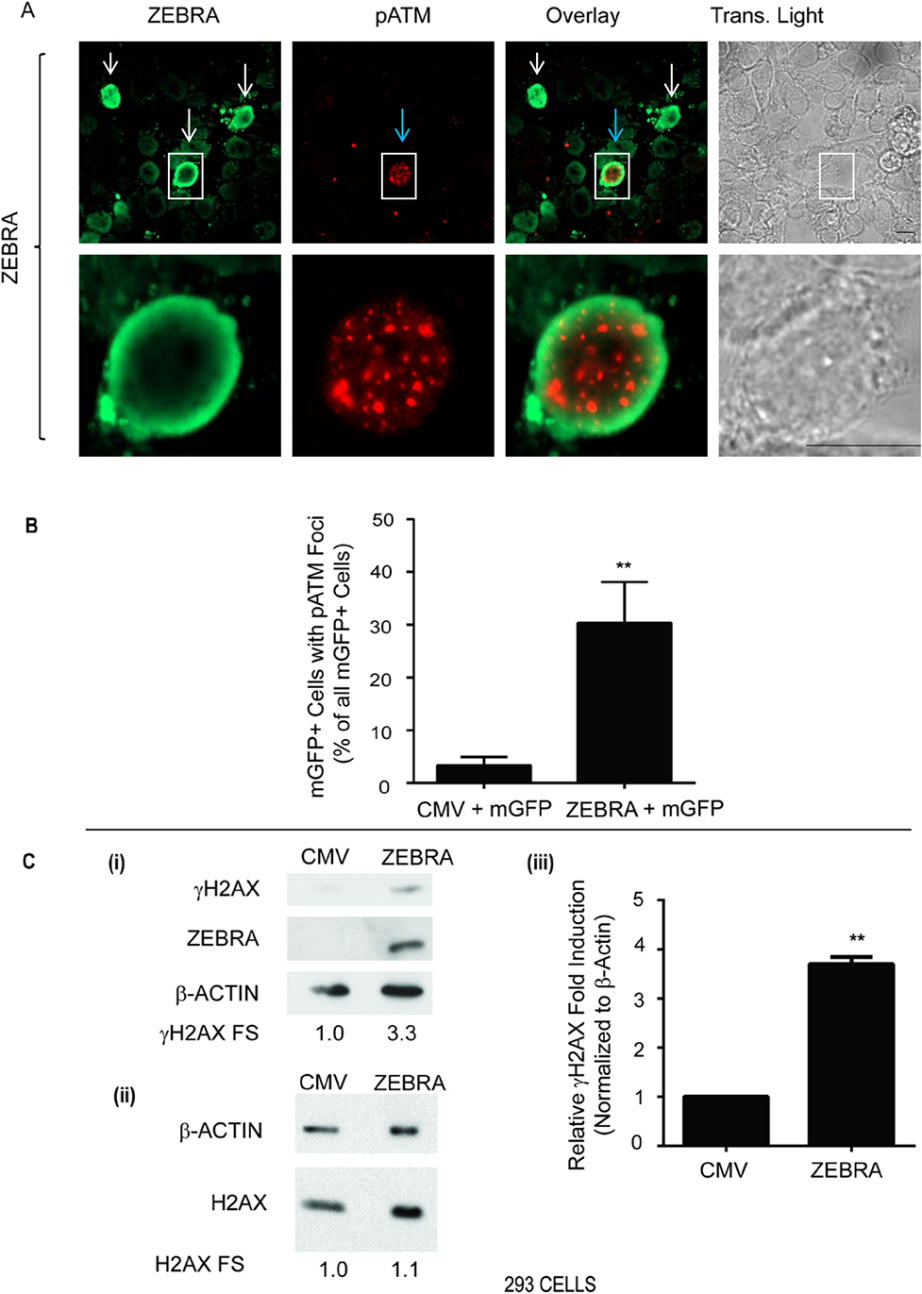
pATM is induced in response to ZEBRA expression in cells lacking EBV. **(A)** 293 cells were transfected with a plasmid expression vector for ZEBRA. Cells were double-stained for ZEBRA and pATM (S1981). White arrows indicate cells expressing ZEBRA. The blue arrow indicates a cell positive for pATM. Scale bar = 10 μm. **(B)** 293 cells were co-transfected with a plasmid expressing a membrane-targeted EGFP-farnesylated construct (mGFP) and an empty vector (CMV) or an expression vector for ZEBRA and stained for pATM (S1981). The percentage of total cells that were positive for both pATM and mGFP was determined in blinded experiments and the results represented as average percentages (n = 4), ** denotes P<0.005; P = 0.0048 for ZEBRA versus CMV. **(C)** 293 cells were transfected with an empty vector (CMV) or a plasmid expression vector for ZEBRA. Cell lysates were analyzed by immunoblots with antibodies against γH2AX and ZEBRA and β-Actin **(C: i)**, or H2AX and β-Actin **(C: ii).** γH2AX fold stimulation (FS) levels are shown; n = 3, ** denotes P<0.005; P = 0.0014 for γH2AX FS in CMV versus ZEBRA 293 samples **(C: iii)**.

**Fig 10:**
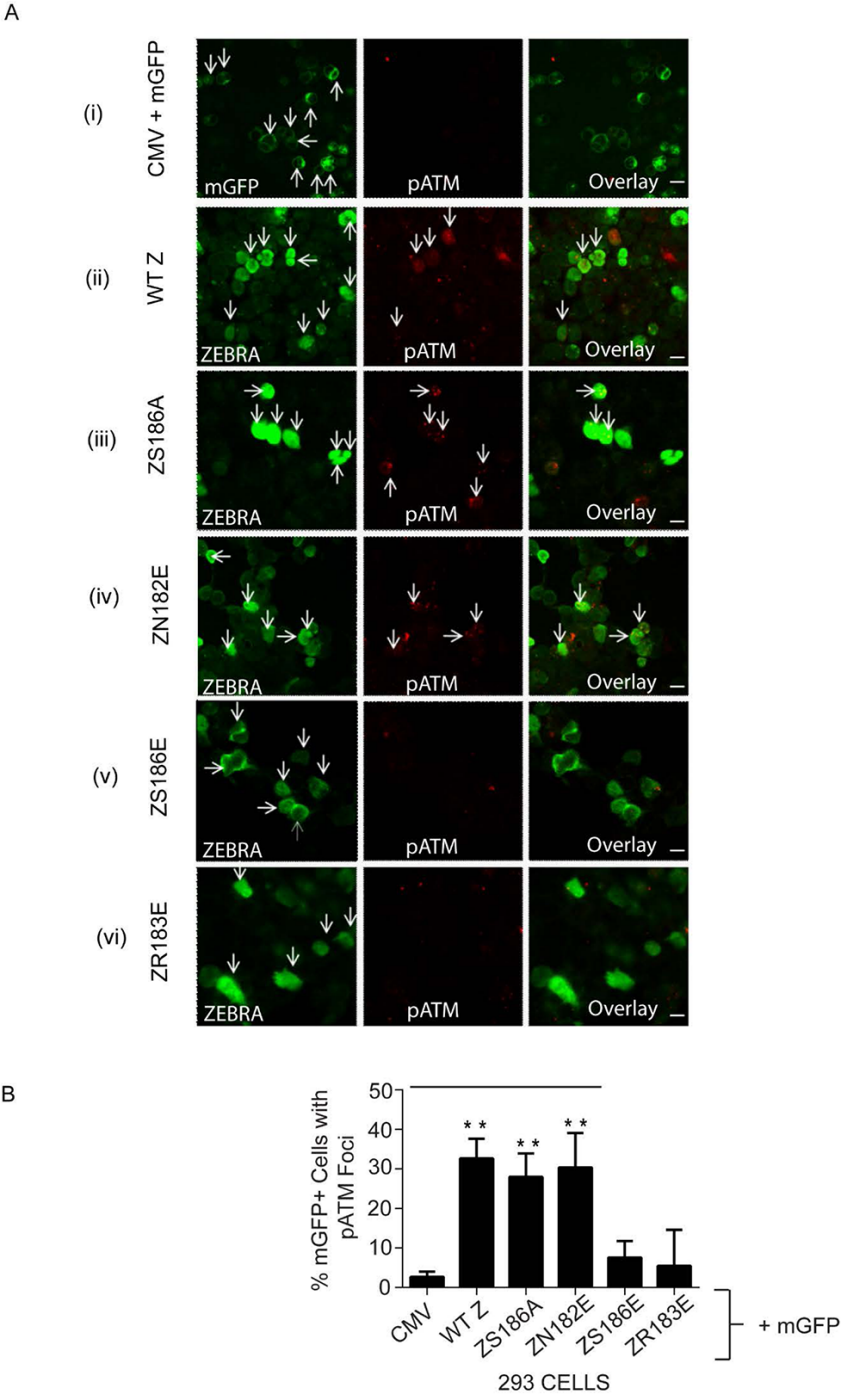
pATM is induced in response to expression of WT ZEBRA, and ZEBRA mutants Z(S186A), and Z(N182E) in 293 cells. **(A)** 293 cells co-transfected with an empty vector (CMV) and a membrane-targeted EGFP-farnesylated construct (mGFP) **(A: i)** or singly transfected with expression vectors for ZEBRA **(A: ii)**, Z(S186A) **(A: iii)**, Z(N182E) **(A: iv)**, Z(S186E) **(A: v)**, or Z(R183E) **(A: vi)** were fixed and double-stained for ZEBRA and pATM (S1981); Arrows indicate transfected cells expressing transfected genes or pATM or transfected genes and pATM. Detector settings were kept constant during confocal image acquisition. **(B)** 293 cells were fixed and stained for pATM (S1981) after co-transfection with a membrane-targeted EGFP-farnesylated construct (mGFP) and an empty vector (CMV), or expression vectors for ZEBRA, or Z(S186A), or Z(N182E), or Z(S186E), or Z(R183E). The percentage of total cells that were positive for both pATM and mGFP was determined in blinded experiments (n = 4). **P<0.01; P = 0.0001 for WTZ vs. CMV, 0.00017 for Z(S186A) vs. CMV, and 0.0013 for Z(N182E) versus CMV.

pATM foci were detected in a significant proportion (about 30%) of ZEBRA-positive 293 cells (Fig 9A and 9B; Fig 10A: ii). We co-transfected 293 cells with CMV and mGFP plasmids or ZEBRA and mGFP plasmids and quantified the proportion of mGFP-positive cells with pATM foci in each sample, in an observer-blinded fashion (Fig 9B). With this blinded approach, there were consistently greater proportions of pATM-positive cells in ZEBRA-transfected cells (Fig 9B). γH2AX was also detected by immunoblot in EBV-negative 293 cells expressing ZEBRA (Fig 9C: i and 9D). These data showed that ZEBRA expression is sufficient for induction of pATM and γH2AX, biochemical markers of DNA damage signaling.

Induction of DNA damage signaling may lead to apoptosis or perturbation of cell cycle progression. Cells expressing ZEBRA were not preferentially stained with 7-aminoactinomycin D (7AAD), which stains apoptotic and dead cells (S4 Fig). These data indicate that cells expressing ZEBRA do not undergo apoptosis or cell death.

### Single amino-acid substitutions in the DNA binding domain of ZEBRA affect its capacity to induce ATM phosphorylation

Some viral transcription factors induce DNA damage in a DNA-binding-dependent manner. We therefore studied ZEBRA mutants with single amino-acid substitutions within the DNA recognition domain that affect binding to a high affinity ZEBRA response element (ZRE), ZIIIB, to determine whether ZEBRA’s ability to induce phosphorylated ATM correlated with its DNA binding capacity. We have previously studied DNA binding and lytic cycle activation properties of ZEBRA mutants, including Z(R179A), Z(S186A), Z(S186E), Z(N182E), and Z(R183E) (Table 1 and [48]). Z(R179A) activates expression of Rta but not EA-D or late lytic proteins when expressed in 2089 EBV-positive cells (Table 1). Z(S186A), Z(S186E), Z(N182E), and Z(R183E) mutants are defective in EBV lytic cycle activation. Of these defective mutants, only Z(S186A) binds to the ZIIIB ZEBRA binding DNA site (Table 1; [48]). Although Z(N182E) is defective in binding ZIIIB sites, it binds to AP1 DNA sequences comparably to wild type ZEBRA (S5 Fig).

**Table 1:** Summary of DNA binding activity, EBV lytic gene activation, and DNA damage signaling phenotypes of ZEBRA mutants compared to wild-type ZEBRA.

Protein	Binding to ZIIIB sequence	*BRLF1* gene activation	*BMRF1* gene activation	*BFRF3* gene activation	pATM foci
WT ZEBRA	+	+	+	+	+
Z(R179A)	+	+	-	-	+
Z(S186A)	+	-	-	-	+
Z(N182E)	-	-	-	-	+
Z(S186E)	-	-	-	-	-
Z(R183E)	-	-	-	-	-

**Fig 11:**
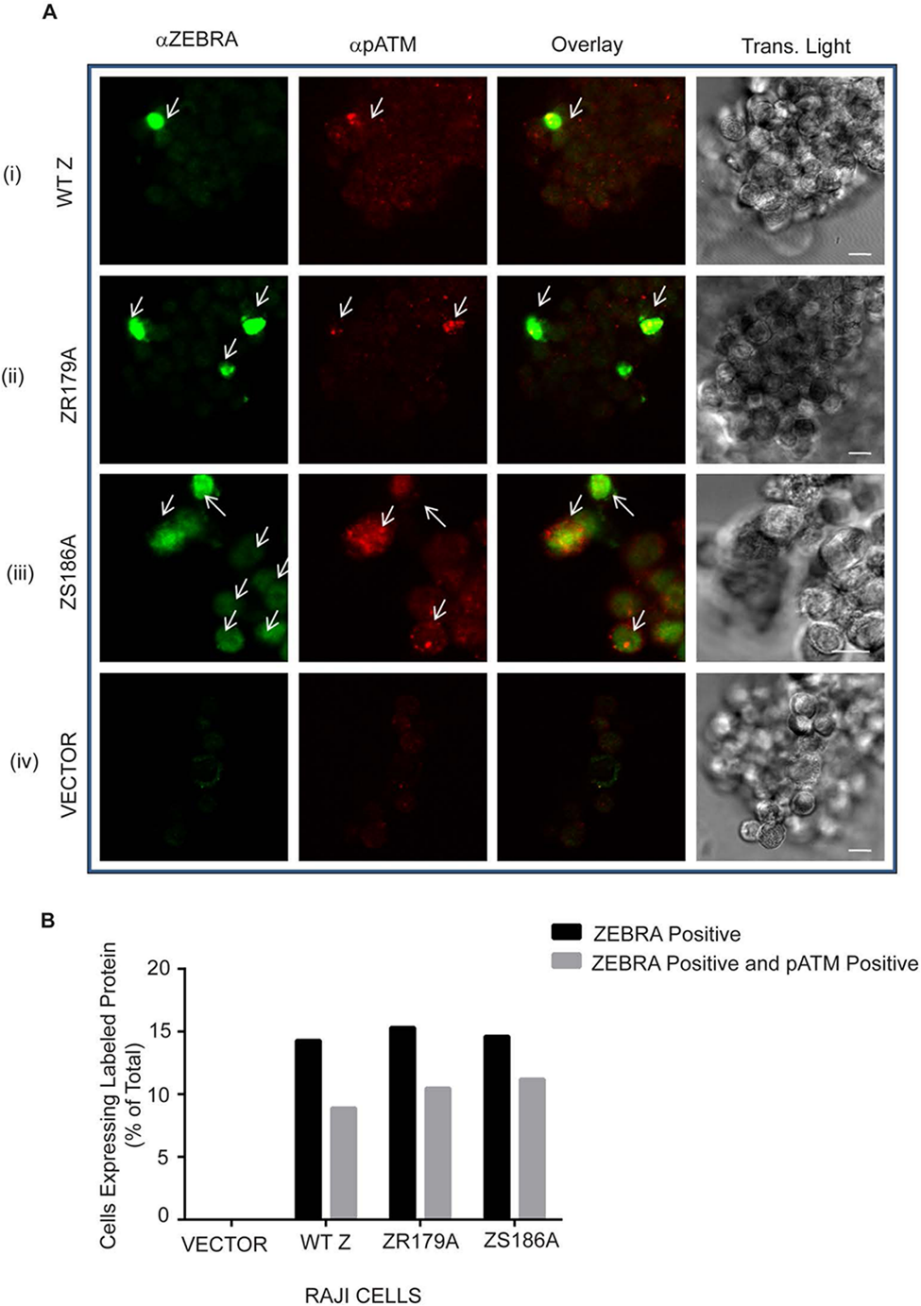
ATM is phosphorylated upon expression of WT ZEBRA, and ZEBRA mutants Z(R179A), and Z(S186A) in Raji cells. **(A)** Raji cells infected with an empty vector lentivirus (V), or lentiviruses expressing the wild-type ZEBRA gene **(A: i)**, Z(R179A) **(A: ii)**, or Z(S186A) **(A: iii)** were fixed and double stained for ZEBRA and pATM. Detector settings were kept constant during confocal image acquisition. Scale bar = 10 μm. **(B)** The percentage of total cells that were positive for ZEBRA or ZEBRA and pATM was obtained.

Capacities for DNA binding and activation of EBV lytic genes by WT and mutant ZEBRA were determined previously [48]. Generation of pATM foci was determined by immunofluorescence assays, in 293 cells, as described in Fig 11.

+ denotes proficiency, relative to WT, in performing the respective activity.

- denotes deficiency, relative to WT, in performing the respective activity.

We compared the relative ability of Z(R179A), Z(S186A), Z(S186E), Z(N182E), and Z(R183E) mutants to activate pATM foci relative to wild type ZEBRA. Expression of ZEBRA mutants was similar to that of wild type ZEBRA (S6 Fig). As seen with wild-type ZEBRA (Fig 9A and 9B and Fig 10A:ii), the Z(S186A) (Fig 10A:iii and 10B) and Z(N182E) (Fig 10A:iv and 10B) mutants induced pATM foci in a significant proportion of 293 cells relative to the negative control (Fig 10B). Foci of pATM were also detected in EBV-positive Raji cells expressing the Z(R179A) (Fig 11A:ii) and Z(S186A) (Fig 11A:iii) mutants. However, the proportion of pATM positive cells in response to expression of DNA binding defective mutants Z(S186E) (Fig 10A: v and 10B) and Z(R183E) was not significantly different from negative control cells (Fig 10A: i and 10B). These data provide additional evidence that ATM phosphorylation in response to expression of ZEBRA is not dependent on ZEBRA’s ability to activate the EBV lytic cycle, but is related to its ability to bind DNA.

### ZEBRA co-localizes with HP1β, a heterochromatin associated protein involved in DNA damage signaling, in electron-dense regions of the nucleus

**Fig 12:**
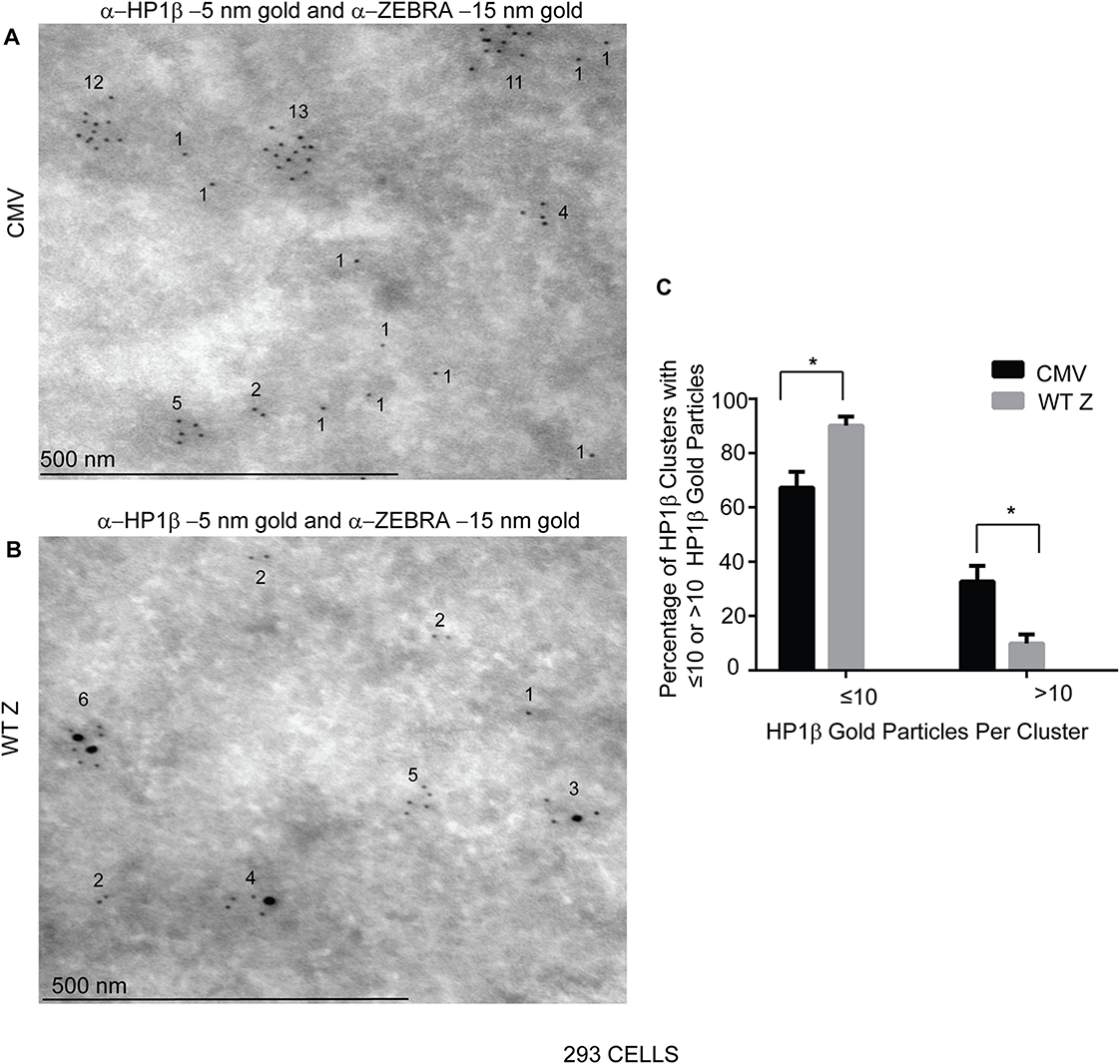
ZEBRA localizes to electron dense regions of the nucleus containing HP1β. 293 cells were transfected with **(A)** an empty vector (CMV), or **(B)** a ZEBRA expression vector, fixed and processed for immunogold labeling with antibodies against ZEBRA and Protein A-conjugated 15 nm gold particles and HP1β and Protein A-conjugated 5 nm gold particles. The percentage of HP1β clusters with ≤10 or >10 5nm gold particles was determined and represented as means (n = 2), 20 nuclei sections were photographed and scored for the presence of each marker with Cell Profiler software. Scale bar = 10 μm.

We have previously shown that ZEBRA localizes to electron dense regions of the nucleus, which are known sites of heterochromatin [49]. To explore a possible mechanism by which ZEBRA may interact with DNA damage signaling pathways, we examined its intra-nuclear distribution relative to HP1β, a heterochromatin associated protein linked to ATM phosphorylation [31]. Immuno-electron microscopy with antibodies conjugated to gold particles showed HP1β localization in numerous foci within electron dense regions of 293 cells (Fig 12). ZEBRA co-localized with HP1β in electron dense regions of the nucleus in EBV- negative 293 cells (Fig 12A and 12B). In the presence of ZEBRA, there were significantly fewer HP1β specific gold particles in electron dense regions of the nucleus (Fig 12C). This finding suggests that ZEBRA may displace HP1β from its binding sites on cellular chromatin (see Discussion).

## Discussion

We show here that activation of DNA damage response signaling proteins can be elicited in the early phase of the EBV lytic cycle and in the absence of EBV DNA replication. Our findings raised two questions: 1) which viral factors are responsible for induction of DNA damage signaling during the early phase of the EBV lytic cycle? 2) What is the function of DNA damage signaling activation associated with the early phase of the EBV lytic cycle? We addressed these questions by investigating the role of early lytic proteins, ZEBRA, BGLF4, BGLF5, and BALF2, in inducing markers of DNA damage signaling and by exploring how the EBV lytic cycle is affected by DNA damage signaling.

### The role of early EBV proteins in inducing DNA damage signaling during the EBV lytic cycle

Although several EBV proteins, including BGLF4 (Fig 3 and [26]), BGFL5 [34], and BALF2 [25], are sufficient to induce markers of DNA damage signaling in EBV-negative cells, we find that these proteins are not necessary for activation of foci of pATM during the EBV lytic cycle (Fig 2B and 2C and Fig 6). pATM foci were detected in ΔG4ΔG5 cells lacking both BGLF4 and BGLF5, upon lytic reactivation by expression of ZEBRA (Fig 2B and 2C). In Raji cells, lacking BALF2, γH2AX (Fig 4A) and pATM foci (Fig 6A; i) were induced in cells lytically reactivated by treatment with TPA.

One scenario that may account for the dispensability of BGLF4, BGLF5, BALF2 in activation of pATM or γH2AX hinges on the roles these proteins play in replication. In the cell systems we studied, BGLF4, BGLF5, or BALF2 were not required for inducing DNA damage signaling events and cells were predominantly in the pre-replicative phase of EBV. The majority of lytically reactivated ΔG4ΔG5 cells did not have replication compartments (Fig 2B and 2C). In Raji cells, due to the absence of the single stranded DNA binding replication protein, BALF2, there was no evidence of EBV DNA replication upon activation of the EBV lytic cycle (Fig 5). It is therefore possible that these lytic proteins may not be necessary for DNA damage signaling in the pre-replicative stages of EBV but may be important for DNA damage signaling induced during or after lytic DNA replication.

Functional redundancy of early lytic proteins in pATM activation may explain why BGLF4, BGLF5, and BALF2 were not necessary for activation of markers of DNA damage signaling during the EBV lytic cycle. BRLF1, an EBV transcriptional activator, has been shown to induce pATM and γH2AX [50]. Additionally, we now show that expression of ZEBRA can also induce pATM (Fig 9 and Fig 10A: i) and γH2AX (Fig 9C) in EBV-negative 293 cells. Thus other early EBV proteins may function to induce DNA damage signaling in the absence of BGLF4, BGLF5, or BALF2 during the EBV lytic cycle.

### Induction of DNA damage signaling markers during the early phase may promote progression of the EBV lytic cycle

The detection of foci of phosphorylated ATM exclusively in EBV-positive cells expressing lytic proteins (Figs 1, 2 and 6) led us to conclude that DNA damage signaling is activated downstream of lytic reactivation. Although ATM kinase activity was not sufficient for EBV reactivation (Fig 7), it was necessary for maximal expression of ZEBRA and EA-D (Fig 8). These data confirm results in the EBV-positive gastric carcinoma AGS-Akata cell line which established a role for ATM in EBV reactivation [8] and show that the requirement for ATM kinase activity is not cell-specific or inducing agent-specific. ATM likely plays a role in enhancing expression of early lytic EBV genes, as previously described [8], and not just viral DNA replication as recently proposed [9]. We therefore propose a model whereby induction of DNA damage signaling is important for progression of the EBV lytic cycle.

### A proposed model for the roles and mechanisms of DNA damage signaling induction during progression of the EBV lytic cycle

**Fig 13:**
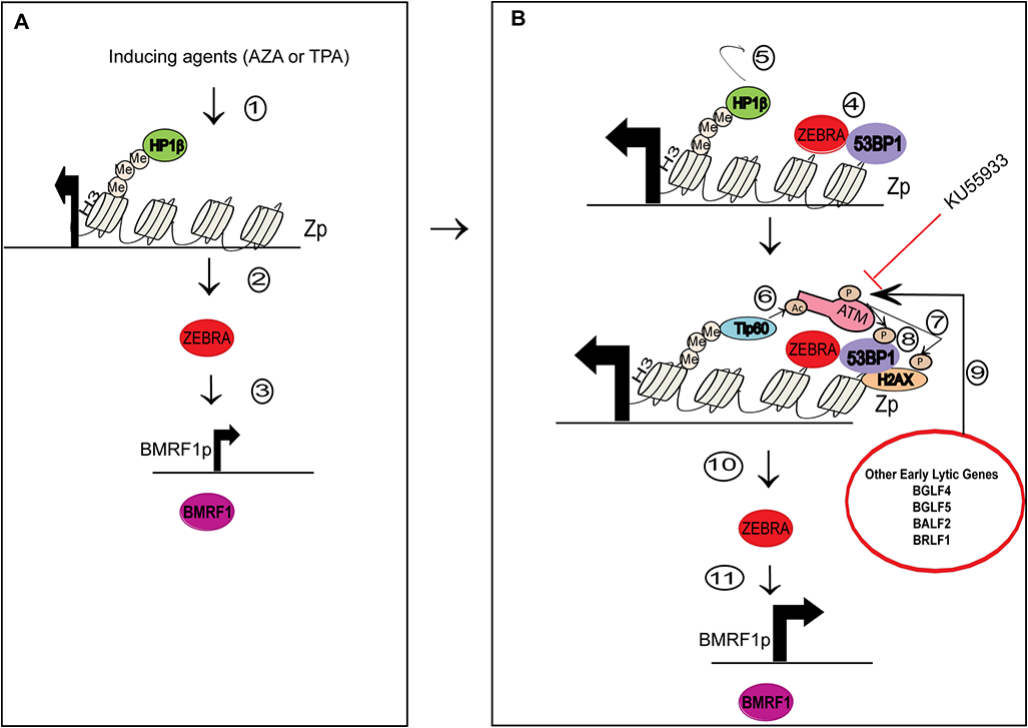
Proposed model for the role of ZEBRA in induction of ATM phosphorylation during the EBV lytic cycle. The model has two broad phases of lytic induction: A) Initial activation of the *BZLF1* promoter (Zp) following induction of the lytic cycle, and B) Further activation of Zp through autoactivation [51] by ZEBRA and induction of ATM phosphorylation and DNA damage signaling by ZEBRA and other early lytic proteins. 1) Inducing agents such as AZA in HH514-16 cells or TPA in Raji cells target Zp which is epigenetically repressed by modified histones such as H3K9Me3 that recruit HP1β [52]. 2) ZEBRA, expressed at low levels 3) stimulates target promoters such as the BMRF1p resulting in expression of EA-D. 4) ZEBRA binds its own promoter where it associates with 53BP1 [53] and 5) and displaces HP1β from H3K9Me. 6) This process allows recruitment of Tip60 which acetylates and activates ATM [54]. 7) ATM phosphorylates H2AX and 8) 53BP1. 9) Other EBV lytic proteins including BGLF4, BGLF5, BALF2, and BRLF1, which are early lytic gene products, may also contribute to ATM kinase activation. ATM kinase activity leads to maximal autoactivation of the ZEBRA promoter leading to 10) increased levels of ZEBRA and 11) increased levels of BMRF1. The ATM inhibitor KU55933 prevents maximal activation of ZEBRA and BMRF1 promoters.

In our model of DNA damage signaling induction during the EBV lytic cycle, we propose that inducing agents such as AZA and TPA initially induce low levels of ZEBRA in target cells which activate target promoters such as the BMRF1 promoter to express low levels of EA-D (Fig 13A; Steps 1–3). ZEBRA is known to activate its own promoter during the EBV lytic cycle [51]. Therefore, the inhibition of maximal ZEBRA and EA-D expression in the presence of the ATM kinase inhibitor, KU55933, (Fig 8) may be explained by a requirement of ATM kinase activity for auto-activation of the ZEBRA promoter (Zp) by ZEBRA (Fig 13B). Other explanations are that the ability of inducing agents to stimulate the Z promoter is enhanced by ATM kinase activity or that ATM enhances ZEBRA or mRNA stability.

The finding that ZEBRA mutants Z(S186E) and Z(R183E), which are deficient in DNA binding (Table 1), were also deficient in inducing foci of ATM phosphorylation (Fig 10) points to a possible role for DNA or chromatin interaction in ZEBRA-induced DNA damage signaling. Z(N182E), a ZEBRA mutant which cannot bind ZIIIB sites (Table 1 and [48]) but binds AP1 sequences comparably to wild type ZEBRA and Z(S186A) (S5 Fig), induced foci of phosphorylated ATM unlike other ZIIIB binding deficient ZEBRA mutants used in this study (Fig 10). Since ZEBRA has the capacity to bind to a heterogeneous group of DNA sequences, including AP1 sites, it is likely that the ability of ZEBRA to induce phosphorylation of ATM, in EBV-negative cells, based on its interactions with DNA, chromatin, or chromatin modifying proteins, may depend on its capacity to bind DNA at AP1 sites or other cellular DNA sequences.

The ATM-mediated pathway of DNA damage signaling can be activated independent of DNA lesions by stable association of DNA repair proteins with chromatin [55] and by chromatin remodeling [10]. ZEBRA may rely on its DNA binding activity to achieve functional proximity with proteins, such as 53BP1[53], which associate with chromatin and are involved in DNA damage signaling. Therefore, a plausible model for ZEBRA-induced ATM phosphorylation may involve interaction between ZEBRA and 53BP1 (Fig 13B; Step 4) at ZEBRA target sites on viral DNA and cellular DNA. ZEBRA may promote stable association of 53BP1 with chromatin and subsequent recruitment and activation of ATM and its substrates (Fig 13B Step 7 and 8).

ZEBRA binds to methylated DNA on viral DNA promoters [56] that contain epigenetically repressive histones, such as H3K9Me [52, 57]. ZEBRA may also bind specifically to methylated DNA on cellular chromatin. Repressive histone marks such as H3K9Me are associated with HP1 β and DNA methylation [58, 59]. This may explain why ZEBRA co-localizes with HP1β on heterochromatin (Fig 13). We noted decreased labeling of HP1 β in cells expressing ZEBRA. ZEBRA may displace HP1 β from electron dense regions of the nucleus (Fig 12). Displacement of HP1β from chromatin in the earliest phase of ATM-dependent DNA damage signaling may recruit the Tip60 acetyltranferase to trimethylated lysine 9 on Histone 3 (H3K9Me). This process amplifies subsequent activation of the ATM-mediated DNA damage signaling pathway [31, 32, 54, 60–62]. Therefore, during the EBV lytic cycle the localization of ZEBRA to genetically repressed chromatin on viral or cellular DNA (Fig 13B) leads to chromatin changes that may displace HP1 β from H3K9Me (Fig 13B; Step 5) allowing for Tip 60 recruitment (Fig 13B; Step 6) and subsequent activation of ATM kinase and phosphorylation of ATM substrates (Fig 13B; Step 7 and 8). Further studies are required to investigate displacement of HP1β by ZEBRA and the involvement of HP1β in ZEBRA’s induction of DNA damage signaling.

Several viral lytic proteins expressed during the early phase of the lytic cycle may cooperate with ZEBRA to induce DNA damage signaling (Fig 13B; step 9). Both ZEBRA and its target genes are maximally activated in the presence of ATM kinase activity (Fig 13B; steps 10 and 11). Our findings showing that induction of markers of DNA damage signaling is not contingent on EBV DNA replication provide a novel perspective on the role of the early phase of the EBV lytic cycle in activating DNA damage responses. Our data support a role for DNA damage signaling in promoting transcriptional activation of viral genes during the EBV lytic cycle.

## Materials and Methods

### Cell Lines

The 293 cell line, a human embryonic kidney (HEK) cell line immortalized by the early region of adenovirus [63], was obtained from the American Type Culture Collection (Cat #: CRL-1573). The 2089 cell line is a 293 cell line stably transfected with a bacmid containing the full-length EBV genome and a hygromycin B-resistance gene [64]. The ΔG4ΔG5 cell line is a 293 cell line stably transfected with a bacmid containing an EBV genome which does not express BGLF4 or BGLF5 proteins due to replacement of a large part of the *BGLF4* gene and *BGLF5* gene promoter with a kanamycin resistance gene cassette [65]. 293, 2089, and ΔG4ΔG5 cells were maintained in DMEM complete media, supplemented with 8% fetal bovine serum, 50 U/mL penicillin–streptomycin, and 1 μg/mL amphotericin B. 100 μg/mL hygromycin B (Calbiochem) was added to media used to grow 2089 and ΔG4ΔG5 cells for bacmid selection. The HH514-16 cell line, developed in our laboratory, is a subclone of the P3J-HRIK Burkitt lymphoma cell line [66]. The Raji cell line, a Burkitt lymphoma cell line lacking the *BALF2* gene, was obtained from the American Type Culture Collection (Cat #: CCL-86) [67]. Burkitt lymphoma cell lines were cultured in RPM1 1640 supplemented with 8% fetal bovine serum, 50 U/mL penicillin–streptomycin, and 1 μg/mL amphotericin B. Cells were grown at 37°C under 5% CO_2_. 293 T cells were obtained from ATCC (Cat#: CRL- 1126). The HKB5 (hybrid kidney B-cell) cell line is a somatic cell hybrid between the 293 human embryonic kidney cell line and the HH514-16 subclone of the P3J-HR1K line from Burkitt lymphoma. HKB5 cells were obtained from Sam Cho [68, 69].

### EBV lytic cycle induction

Burkitt lymphoma cell lines in logarithmic-phase growth, usually at 48 h after the last subculture, were resuspended at 10^6^/ml in fresh medium and treated with lytic cycle-inducing chemicals. The inducing chemicals 5 μM 5-aza-2’-deoxycytidine (AZA) (Sigma no. 3656) or 20 ng/ml TPA (Calbiochem no. 524400) and 3 mM sodium butyrate (NaB) (Sigma no. B5887) were used in HH514-16 or Raji cells, respectively. Cells were treated with inducing agents for 24 h. 2089 and ΔG4ΔG5 cells and were induced into the lytic cycle by transfection with a plasmid containing the ZEBRA gene. Transfections were conducted for 32 hours.

### Treatment of cells with DNA damaging reagents and ATM kinase inhibitor

Cells were irradiated with a ^137^Cs source at a dose rate of 1.46 Gy per min or treated with 30 μg/ml of camptothecin (Sigma C9911). Cells were treated with KU55933 (KuDos pharmaceuticals) at a final concentration of 15 μM to inhibit ATM kinase activity.

### Expression constructs, transfection, and lentivirus transduction

The plasmids pHD1013/Z, pHD1013/Z(S186A), pHD1013/Z(S186E), pHD1013/Z(R179A), pHD1013/Z(R183E), pHD1013/Z(N182E) were described previously [48, 70]. The construct expressing BGLF4 was provided by Mei-Ru Chen [71]. The membrane-targeted EGFP-farnesylated construct (mGFP) was provided by Tony Hunter. For transfection experiments cells were transfected with plasmid DNA using DMRIE-C reagent (Invitrogen). After 8 h the transfection reagent was replaced with growth media and cells were incubated for another 24 hours.The lentivirus construct P3465V was kindly provided by Bill Sugden. PCR fragments of wild-type ZEBRA or ZEBRA mutants with BamHI and EcoRI restriction sites at 5’ and 3’ ends, respectively, were created from respective pHD1013 plasmids. PCR fragments were digested with BamH1 and EcoRI and ligated into the P3465V construct digested with BamH1 and EcoRI. For packaging of lentiviruses, transient transfection experiments in 293T cells were undertaken with lenti-Rev, lenti-Gag/pol, lenti-Tet, and lenti-vsvG plasmid constructs (kindly provided by Stephen Elledge) and respective lentiviral DNA at the ratio of 1:1:1:2:10 and the DMRIE-C reagent (Invitrogen) transfection protocol. Lentivirus medium was collected after 52 and 72 hours and centrifuged. After centrifugation supernatants were collected and immediately used to infect target cells. For lentivirus transduction cells were infected with P3465 lentivirus containing the respective gene upstream of IRES-GFP or no gene for empty vector controls by culturing cells with mixture of lentivirus supernatant and 8μg/ml Polybrene (Millipore no. TR-1003-G).

### Antibodies

In immunoblot and immunofluorescence experiments, ZEBRA was detected using a rabbit polyclonal antibody (S1605-1)described previously [72]. EA-D was detected using the mouse monoclonal antibody R3.1 [73].Rta and BFRF3 proteins were detected using rabbit polyclonal antisera described previously [48, 74]. pATM (S1981) was detected with a mouse monoclonal antibody (Rockland Immunochemicals #200-301-400). γH2AX (S139) was detected with a mouse monoclonal antibody (Millipore #05–636). H2AX was detected with a rabbit polyclonal antibody (Millipore #07–627). p53BP1 (S1778) was detected by a rabbit polyclonal antibody (Cell Signaling Technology no. 2675). Mouse antibodies to β-actin (A5316; Sigma) or GAPDH were used to control for protein loading. The following secondary antibodies, used in immunofluorescence experiments, were purchased from Jackson ImmunoResearch Laboratories, Inc.: FITC-conjugated sheep anti-mouse IgG (#515-095-062), Texas Red-conjugated donkey anti-rabbit IgG (#711-075-152), FITC-conjugated donkey anti-goat IgG (#705-095-147). Alexa Fluor 647-anti-mouse IgG (#715-605-150). Alexa Fluor 647-anti-rabbit IgG (#711-605-152).

### Indirect immunofluorescence

293, 2089, or ΔG4ΔG5 cells were grown on glass coverslips and transfected for 32 hours with respective plasmid DNA using DMRIE-C reagent (Invitrogen). Cells were fixed with methanol for 30 min at − 20°C, washed with PBS and incubated in blocking solution (10% human serum in PBS) for 1 h at room temperature and stained with primary antibody diluted in blocking solution for 1 h at room temperature in humidified chambers. Cells were washed with PBS, then incubated with secondary antibody diluted 1:200 in blocking solution for 1 h at room temperature. Cells were washed with PBS, briefly rinsed in distilled H_2_O to remove salts, and mounted on glass slides using Vectashield mounting media (Vector Laboratories). HH514-16 and Raji cells were fixed for 20 min with 4% paraformaldehyde after treatment with inducing agents or other chemicals, or lentivirus transduction. For permeabilized cells were incubated for 5 minutes in 0.1% TritonX-100 twice and washed with PBS between incubations. The staining protocol described above was used in Raji and HH514-16 cells. After staining, Raji and HH514-16 cells were pelleted and resuspended in 300 μl of liquefied glycerol gelatin aqueous slide mounting medium (Sigma) then pipetted onto glass slides and covered with cover slips. A Zeiss LSM510 confocal laser scanning microscope was used to obtain digital images of fluorescence and transmitted light. Excited fluorophore emissions were collected either individually or simultaneously under detection settings that reduced cross-talk between the green and red channels to non-detectable levels. For quantification approximately 300 cells or 100 cells were counted per experiment in EBV-positive cell lines or EBV-negative cell lines. The percentage of ZEBRA or ZEBRA mutant tranfected cells with pATM foci was obtained in blinded assays where the operator was blinded to sample identities during data collection. In these blinded assays, mGFP was co-transfected with respective DNA and used as a surrogate marker for transfection.

### Flow Cytometry

7-aminoactinomycin D (7AAD) (Calbiochem Cat. # 129935) was used to stain apoptotic and dead cells per previously established protocols [75]. Briefly, 10^6^ cells cotransfected with an mGFP plasmid and emty vector or were stained with 20 μg/ml of 7AAD solution for 20 minutes at 4°C in the dark. The supernatant was removed and cells were resuspended in 500 μl 2% paraformaldehyde solution (Sigma). Unstained, fixed, cells were used as negative controls. 7AAD stained samples were analyzed on a FACSCalibur flow cytometer (Beckton Dickinson, Mountain View, CA) within 30 minutes of fixation. Data on 50,000 cells was acquired and processed using FlowJo software. Gates for low or high 7AAD staining were set using camptothecin-treated versus untreated cells.

### Real-time RT-PCR and quantitative PCR

Total RNA was isolated using an RNeasy kit with on-column DNase digestion (Qiagen). The relative transcript levels of the *bzlf1* gene were determined using real-time RT-PCR with BZLF1 specific primers (forward, TACAAGAATCGGGTGGCTTC; reverse, GCACATCTGCTTCAACAGGA) using the iScript SYBR green RT-PCR kit (Bio-Rad). Serial dilutions of gel-purified BZLF1 cDNA fragments were used to generate standard curves to quantitate the BZLF1. Individual samples were assayed in triplicate; in two biological replicates. Primers have been described previously [76]. Quantitative PCR for detection of viral genome amplification was previously described [77].

### Immunoelectron Microscopy

293 cells were transfected with the pHD1013/Z plasmid bearing the ZEBRA gene or pHD1013 empty vector plasmid using DMRIE-C reagent (Invitrogen). Samples were fixed in 2% paraformaldehyde for 30 min at 4°C, rinsed in PBS, resuspended in 10% gelatin, chilled, trimmed to smaller blocks, and placed in cryoprotectant (2.3 m sucrose) overnight at 4°C. Blocks were transferred to aluminum pins and frozen rapidly in liquid nitrogen. Frozen blocks were trimmed on a Leica cryo-EM UC6 UltraCut, and 75-nm-thick sections were collected using the method of Tokuyasu [78]. Sections were placed on a nickel Formvar/carbon-coated grid and floated in a dish of PBS for immunolabeling. Grids were placed section side down on drops of 0.1 m ammonium chloride for 10 min to quench untreated aldehyde groups, and then blocked with 1% fish skin gelatin in PBS for 20 min. Single labeled grids were incubated with rabbit anti-ZEBRA rabbit IgG (1:50) or anti- HP1β mouse IgG (1:10) (Pierre Chambon’s laboratory) followed by a rabbit anti-mouse bridge (JacksonImmuno). 10 nm Protein A gold (University of Utrecht Medical Center) was used as secondary antibody. Double labeling was done as above fixing in 2% formaldehyde before running the secondary primary antibody. Either 5 nm or 15 nm Protein A gold was used for antibody differentiation. All grids were rinsed in PBS, fixed using 1% glutaraldehyde for 5 min, rinsed again, transferred to a uranyl acetate/methylcellulose drop for 10 min, then collected, and dried. Samples were viewed on a FEI Tencai Biotwin transmission electron microscope at 80 kV. Images were taken using a Morada charge-coupled device camera and iTEM (Olympus) software. The open-source high-throughput image analysis software CellProfiler was used to analyze electron micrographs.

### Electrophoretic Mobility Shift Assays (EMSAs)

EMSAs were conducted as previously described [48]. Briefly, EMSA binding reactions contained 10 μg protein from HKB5/B5 cell extracts, 10 μl 2× buffer (20 mM HEPES, pH 7.5, 100 mM NaCl, 4 mM MgCl2, 5 μM ZnSO4, 1 mM EDTA, 2 mM DTT, 30% glycerol), 500 ng poly(dI-dC) in 0.5 μl, duplex oligonucleotide probe at approximately 0.1 ng in 0.5 μl, and double-distilled H2O to a 20-μl final volume. A probe containing ZEBRA recognition element ZIIIB (TTAGCAA) was made by annealing two oligonucleotides: (5′-GATCGGTACATTAGCAATGCCTG-3′ and 5′-GATCCAGGCATTGCTAATGTACC-3′), which were end labeled with γ-32P by T4 polynucleotide kinase. A probe containing the AP1 heptamer (TGAGTCA) was made by annealing two oligonucleotides: (5’GATCCAGGCA TGAGTCATGTACC3’ and 5’GATCGGTACATGACTCA TGCCTG3’). For supershift reactions, 1 μl to 4 μl of BZ-l monoclonal antibody (MAb), purchased from DAKO, was added to the reaction mixture. Samples were loaded onto a 6% polyacrylamide gel made with 0.5× TBE (Tris-borate-EDTA) buffer. The gel was pre-run for 0.5 h at 225 V in 0.5× TBE buffer before being loaded. After the samples were loaded, the gel was run at 225 V until a bromphenol blue marker lane reached three-fourths down the gel. The gel was vacuum dried onto Whatman paper at 80°C for 1 to 2 h. The gel was exposed to Kodak XAR-5 film overnight.

## Supporting Information

S1 Fig Levels of H2AX do not change in response to treatment of HH514-16 cells with AZA. Cell lysates from HH514-16 cells not treated (UN) or treated with 5-aza-2’-deoxycytidine (AZA) for 6, 16, or 24 hours, used in Fig 4E, were analyzed by immunoblots with antibodies against β-actin and H2AX (S135). The indicated average fold-induction values of H2AX, based on densitometry values of H2AX bands normalized to unphosphorylated β-actin bands in untreated versus AZA samples at each time point, were used in calculations of fold induction values of γH2AX bands normalized to unphosphorylated H2AX in Fig 4F.
(TIF)

S2 Fig The EBV lytic cycle is not activated in response to treatment of Burkitt Lymphoma cells with camptothecin. Cell lysates from **(A)** HH514-16 cells untreated or treated with AZA for 24 hours or camptothecin (CAMPTO) or **(B)** Raji cells untreated or treated with TPA or Camptothecin were analyzed by immunoblots with antibodies against ZEBRA and β-actin. In both panels, camptothecin was washed off after 2 hours of treatment and cells incubated for an additional 22 hours.
(TIF)

S3 Fig pATM is not induced following treatment of Burkitt lymphoma cell lines with AZA, TPA, or PAA in the absence of EBV lytic reactivation. **(A)** Raji cells treated with AZA or HH514-16 cells treated with **(B)** PAA or **(C)** TPA were double-stained for ZEBRA and pATM (S1981).
(TIF)

S4 Fig Expression of ZEBRA in 293 cells does not increase apoptotic and dead cells. 293 cells were transfected with CMV (B, i) or WTZ (B, ii) and a plasmid bearing prenylated GFP that localizes to the membrane (mGFP), as a marker for transfected cells. After 32 hours, cells were treated with 7-Amino-actinomycin D (7AAD) and analyzed by flow cytometry to detect apoptotic (low 7AAD staining) and dead cells (high 7AAD staining). Flourescent activated cell sorting (FACS) plots of cells are shown. The percentages of total GFP positive or negative cells with high, low or no 7AAD staining are shown.
(TIF)

S5 Fig The Z(N182E) mutant binds better than to an AP1 DNA sequence compared to a ZIIIB DNA sequence. HKB5/B5 cells were transfected with Z(N182E), wild type ZEBRA (WTZ), or CMV plasmids, or mock-transfected (Mock). Shown are EMSAs using whole cell extracts of transfected cells. Relative binding of Z(N182E) or WT Z to a radioactive probe containing **A)** a ZIIIB DNA sequence (TTAGCAA) or **B)** AP1 DNA sequence (TGAGTCA) was determined using 1 μl of a monoclonal antibody to ZEBRA (BZ1 MAb). SS denotes super shifted band; NS denotes non-specific band.
(TIF)

S6 Fig Wild-type ZEBRA and ZEBRA mutants are expressed to similar levels in 293 cells. Cell lysates of 293 cells transfected with wild type ZEBRA, ZN182E, ZS186A, ZS186E, or ZR183E mutants were analyzed by immunoblots with antibodies against ZEBRA and β-ACTIN.
(TIF)

## References

[1] Raab-Traub N, Flynn K, Pearson G. The differentiated form of nasopharyngeal carcinoma contains Epstein-Barr virus DNA. International Journal of Cancer. 1987;39(1):25–9. 3025109 10.1002/ijc.2910390106

[2] Old L, Boyse E, Oettgen H, Harven E, Geering G, Williamson B, et al Precipitating antibody in human serum to an antigen present in cultured Burkitt's Lymphoma cells. Proc Natl Acad Sci U S A. 1966;56(6):1699–704. 16591407 10.1073/pnas.56.6.1699 PMC220158

[3] Wolf H, Zur Hausen H, Becker V. EB viral genomes in epithelial nasopharyngeal carcinoma cells. Nature New Biol. 1973;244(138):245–7. 4353684 10.1038/newbio244245a0

[4] Tsai MH, Raykova A, Klinke O, Bernhardt K, Gartner K, Leung CS, et al Spontaneous lytic replication and epitheliotropism define an Epstein-Barr virus strain found in carcinomas. Cell Reports. 2013;5(2):458–70. Epub 2013/10/15. 10.1016/j.celrep.2013.09.012 24120866

[5] Nikitin PA, Yan CM, Forte E, Bocedi A, Tourigny JP, White RE, et al An ATM/Chk2-mediated DNA damage-responsive signaling pathway suppresses Epstein-Barr virus transformation of primary human B cells. Cell Host and Microbe. 2010;8(6):510–22. 10.1016/j.chom.2010.11.004 21147465 PMC3049316

[6] Chaurushiya MS, Weitzman MD. Viral manipulation of DNA repair and cell cycle checkpoints. DNA Repair. 2009;8(9):1166–76. 10.1016/j.dnarep.2009.04.016 19473887 PMC2725192

[7] Kudoh A, Fujita M, Zhang L, Shirata N, Daikoku T, Sugaya Y, et al Epstein-Barr virus lytic replication elicits ATM checkpoint signal transduction while providing an S-phase-like cellular environment. Journal of Biological Chemistry. 2005;280(9):8156–63. 15611093 10.1074/jbc.M411405200

[8] Hagemeier SR, Barlow EA, Meng Q, Kenney SC. The cellular ataxia telangiectasia-mutated kinase promotes epstein-barr virus lytic reactivation in response to multiple different types of lytic reactivation-inducing stimuli. J Virol. 2012;86(24):13360–70. Epub 2012/09/28. 10.1128/JVI.01850-12 23015717 PMC3503132

[9] Hau PM, Deng W, Jia L, Yang J, Tsurumi T, Chiang AK, et al Role of ATM in the Formation of the Replication Compartment during Lytic Replication of Epstein-Barr Virus in Nasopharyngeal Epithelial Cells. J Virol. 2015;89(1):652–68. Epub 2014/10/31. 10.1128/JVI.01437-14 25355892 PMC4301132

[10] Bakkenist CJ, Kastan MB. DNA damage activates ATM through intermolecular autophosphorylation and dimer dissociation. Nature. 2003;421(6922):499–506. 12556884 10.1038/nature01368

[11] Manke IA, Lowery DM, Nguyen A, Yaffe MB. BRCT Repeats As Phosphopeptide-Binding Modules Involved in Protein Targeting. Science. 2003;302(5645):636–9. 14576432 10.1126/science.1088877

[12] Matsuoka S, Rotman G, Ogawa A, Shiloh Y, Tamai K, Elledge SJ. Ataxia telangiectasia-mutated phosphorylates Chk2 in vivo and in vitro. Proceedings of the National Academy of Sciences of the United States of America. 2000;97(19):10389–94. 10973490 10.1073/pnas.190030497 PMC27034

[13] Yazdi PT, Wang Y, Zhao S, Patel N, Lee EYHP, Qin J. SMC1 is a downstream effector in the ATM/NBS1 branch of the human S-phase checkpoint. Genes and Development. 2002;16(5):571–82. 11877377 10.1101/gad.970702 PMC155356

[14] Gu H, Liang Y, Mandel G, Roizman B. Components of the REST/CoREST/histone deacetylase repressor complex are disrupted, modified, and translocated in HSV-1-infected cells. Proceedings of the National Academy of Sciences of the United States of America. 2005;102(21):7571–6. 15897453 10.1073/pnas.0502658102 PMC1140450

[15] Lomonte P, Thomas J, Texier P, Caron C, Khochbin S, Epstein AL. Functional interaction between class II histone deacetylases and ICP0 of herpes simplex virus type 1. Journal of Virology. 2004;78(13):6744–57. 15194749 10.1128/JVI.78.13.6744-6757.2004 PMC421675

[16] Kofod-Olsen E, Ross-Hansen K, Mikkelsen JG, Höllsberg P. Human herpesvirus 6B U19 protein is a PML-regulated transcriptional activator that localizes to nuclear foci in a PML-independent manner. Journal of General Virology. 2008;89(1):106–16. 18089734 10.1099/vir.0.83224-0

[17] Kofod-Olsen E, Moller JM, Schleimann MH, Bundgaard B, Bak RO, Oster B, et al Inhibition of p53-dependent, but not p53-independent, cell death by U19 protein from human herpesvirus 6B. PLoS ONE. 2013;8(3):e59223 Epub 2013/04/05. 10.1371/journal.pone.0059223 23555634 PMC3608612

[18] Lilley C, Chaurushiya M, Weitzman M. Chromatin at the intersection of viral infection and DNA damage. Biochim Biophys Acta. 2010;1799(3–4):319–27. 10.1016/j.bbagrm.2010.08.007 19616655 PMC2838936

[19] Baer R, Bankier AT, Biggin MD. DNA sequence and expression of the B95-8 Epstein-Barr virus genome. Nature. 1984;310(5974):207–11. 6087149 10.1038/310207a0

[20] Daikoku T, Kudoh A, Fujita M, Sugaya Y, Isomura H, Shirata N, et al Architecture of replication compartments formed during Epstein-Barr virus lytic replication. Journal of Virology. 2005;79(6):3409–18. 15731235 10.1128/JVI.79.6.3409-3418.2005 PMC1075702

[21] Park R, Heston L, Shedd D, Delecluse HJ, Miller G. Mutations of amino acids in the DNA-recognition domain of Epstein-Barr virus ZEBRA protein alter its sub-nuclear localization and affect formation of replication compartments. Virology. 2008;382(2):145–62. 10.1016/j.virol.2008.09.009 18937960 PMC2654287

[22] Henle G, Henle W, Klein G. Demonstration of two distinct components in the early antigen complex of Epstein-Barr virus-infected cells. International Journal of Cancer. 1971;8(2):272–82. 4332368 10.1002/ijc.2910080212

[23] Weitzman MD, Carson CT, Schwartz RA, Lilley CE. Interactions of viruses with the cellular DNA repair machinery. DNA Repair. 2004;3(8–9):1165–73. 15279805 10.1016/j.dnarep.2004.03.018

[24] Weitzman MD, Lilley CE, Chaurushiya MS. Genomes in conflict: maintaining genome integrity during virus infection. Annu Rev Microbiol. 2010;64:61–81. Epub 2010/08/10. 10.1146/annurev.micro.112408.134016 20690823

[25] Fang CY, Lee CH, Wu CC, Chang YT, Yu SL, Chou SP, et al Recurrent chemical reactivations of EBV promotes genome instability and enhances tumor progression of nasopharyngeal carcinoma cells. International Journal of Cancer. 2009;124(9):2016–25. 19132751 10.1002/ijc.24179

[26] Li R, Zhu J, Xie Z, Liao G, Liu J, Chen MR, et al Conserved herpesvirus kinases target the DNA damage response pathway and TIP60 histone acetyltransferase to promote virus replication. Cell Host and Microbe. 2011;10(4):390–400. 10.1016/j.chom.2011.08.013 22018239 PMC3253558

[27] Tarakanova VL, Leung-Pineda V, Hwang S, Yang CW, Matatall K, Basson M, et al γ-Herpesvirus Kinase Actively Initiates a DNA Damage Response by Inducing Phosphorylation of H2AX to Foster Viral Replication. Cell Host and Microbe. 2007;1(4):275–86. 18005708 10.1016/j.chom.2007.05.008 PMC2034359

[28] Chang YH, Lee CP, Su MT, Wang JT, Chen JY, Lin SF, et al Epstein-Barr virus BGLF4 kinase retards cellular S-phase progression and induces chromosomal abnormality. PLoS ONE. 2012;7(6):e39217 Epub 2012/07/07. 10.1371/journal.pone.0039217 22768064 PMC3387188

[29] McFadden K, Luftig MA. Interplay between DNA tumor viruses and the host DNA damage response. Curr Top Microbiol Immunol. 2013;371:229–57. Epub 2013/05/21. 10.1007/978-3-642-37765-5_9 23686238 PMC6707713

[30] Ramasubramanyan S, Osborn K, Flower K, Sinclair AJ. Dynamic Chromatin Environment of Key Lytic Cycle Regulatory Regions of the Epstein-Barr Virus Genome. Journal of Virology. 2012;86(3):1809–19. 10.1128/JVI.06334-11 22090141 PMC3264371

[31] Ayoub N, Jeyasekharan AD, Bernal JA, Venkitaraman AR. HP1-β mobilization promotes chromatin changes that initiate the DNA damage response. Nature. 2008;453(7195):682–6. 10.1038/nature06875 18438399

[32] Ayoub N, Jeyasekharan AD, Venkitaraman AR. Mobilization and recruitment of HP1: a bimodal response to DNA breakage. Cell Cycle. 2009;8(18):2945–50. Epub 2009/08/07. 19657222

[33] Price BD, D'Andrea AD. Chromatin remodeling at DNA double-strand breaks. Cell. 2013;152(6):1344–54. 10.1016/j.cell.2013.02.011 23498941 PMC3670600

[34] Wu CC, Liu MT, Chang YT, Fang CY, Chou SP, Liao HW, et al Epstein-Barr Virus DNase (BGLF5) induces genomic instability in human epithelial cells. Nucleic Acids Research. 2009;38(6):1932–49. 10.1093/nar/gkp1169 20034954 PMC2847232

[35] El-Guindy A, Lopez-Giraldez F, Delecluse HJ, McKenzie J, Miller G. A Locus Encompassing the Epstein-Barr Virus bglf4 Kinase Regulates Expression of Genes Encoding Viral Structural Proteins. PLoS Pathog. 2014;10(8):e1004307 Epub 2014/08/29. 10.1371/journal.ppat.1004307 25166506 PMC4148442

[36] Nyormoi O, Thorley Lawson DA, Elkington J, Strominger JL. Differential effect of phosphonoacetic acid on the expression of Epstein Barr viral antigens and virus production. Proceedings of the National Academy of Sciences of the United States of America. 1976;73(5):1745–8. 179098 10.1073/pnas.73.5.1745 PMC430377

[37] Zhang CX, Decaussin G, Daillie J, Ooka T. Altered expression of two Epstein-Barr virus early genes localized in BamHI-A in nonproducer Raji cells. Journal of Virology. 1988;62(6):1862–9. 2835494 10.1128/jvi.62.6.1862-1869.1988 PMC253267

[38] Avemann K, Knippers R, Koller T, Sogo JM. Camptothecin, a specific inhibitor of type I DNA topoisomerase, induces DNA breakage at replication forks. Molecular and Cellular Biology. 1988;8(8):3026–34. 2850477 10.1128/mcb.8.8.3026-3034.1988 PMC363528

[39] Rogakou EP, Nieves-Neira W, Boon C, Pommier Y, Bonner WM. Initiation of DNA fragmentation during apoptosis induces phosphorylation of H2AX histone at serine 139. Journal of Biological Chemistry. 2000;275(13):9390–5. 10734083 10.1074/jbc.275.13.9390

[40] Banin S, Moyal L, Shieh SY, Taya Y, Anderson CW, Chessa L, et al Enhanced phosphorylation of p53 by ATM in response to DNA damage. Science. 1998;281(5383):1674–7. 9733514 10.1126/science.281.5383.1674

[41] Canman CE, Lim DS, Cimprich KA, Taya Y, Tamai K, Sakaguchi K, et al Activation of the ATM kinase by ionizing radiation and phosphorylation of p53. Science. 1998;281(5383):1677–9. 9733515 10.1126/science.281.5383.1677

[42] Zhang Y, Adachi M, Zou H, Hareyama M, Imai K, Shinomura Y. Histone deacetylase inhibitors enhance phosphorylation of histone H2AX after ionizing radiation. International Journal of Radiation Oncology Biology Physics. 2006;65(3):859–66. 16751067 10.1016/j.ijrobp.2006.03.019

[43] Saito S, Goodarzi AA, Higashimoto Y, Noda Y, Lees-Miller SP, Appella E, et al ATM mediates phosphorylation at multiple p53 sites, including Ser46, in response to ionizing radiation. Journal of Biological Chemistry. 2002;277(15):12491–4. 11875057 10.1074/jbc.C200093200

[44] Bai L, Merchant JL. ATM phosphorylates ZBP-89 at Ser202 to potentiate p21(waf1) induction by butyrate. Biochemical and Biophysical Research Communications. 2007;359(3):817–21. 17560543 10.1016/j.bbrc.2007.05.197 PMC1994773

[45] Rogakou EP, Pilch DR, Orr AH, Ivanova VS, Bonner WM. DNA double-stranded breaks induce histone H2AX phosphorylation on serine 139. Journal of Biological Chemistry. 1998;273(10):5858–68. 9488723 10.1074/jbc.273.10.5858

[46] Hickson I, Zhao Y, Richardson CJ, Green SJ, Martin NMB, Orr AI, et al Identification and characterization of a novel and specific inhibitor of the ataxia-telangiectasia mutated kinase ATM. Cancer Research. 2004;64(24):9152–9. 15604286 10.1158/0008-5472.CAN-04-2727

[47] Countryman J, Miller G. Activation of expression of latent Epstein-Barr herpesvirus after gene transfer with a small cloned subfragment of heterogeneous viral DNA. Proceedings of the National Academy of Sciences of the United States of America. 1985;82(12):4085–9. Epub 1985/06/01. 2987963 10.1073/pnas.82.12.4085 PMC397939

[48] Heston L, El-Guindy A, Countryman J, Dela Cruz C, Delecluse HJ, Miller G. Amino acids in the basic domain of Epstein-Barr virus ZEBRA protein play distinct roles in DNA binding, activation of early lytic gene expression, and promotion of viral DNA replication. Journal of Virology. 2006;80(18):9115–33. 16940523 10.1128/JVI.00909-06 PMC1563939

[49] Park R, Wang'ondu R, Heston L, Shedd D, Miller G. Efficient induction of nuclear aggresomes by specific single missense mutations in the DNA-binding domain of a viral AP-1 homolog. Journal of Biological Chemistry. 2011;286(11):9748–62. 10.1074/jbc.M110.198325 21233201 PMC3059024

[50] Chen Y, Chen Y, Tsai W, Ko Y, Chen J, Lin S. The Epstein-Barr virus replication and transcription activator, Rta/BRLF1, induces cellular senescence in epithelial cells. Cell Cycle. 2009;8(1):58–65. 19098430 10.4161/cc.8.1.7411

[51] Flemington E, Speck SH. Autoregulation of Epstein-Barr virus putative lytic switch gene BZLF1. Journal of Virology. 1990;64(3):1227–32. 2154606 10.1128/jvi.64.3.1227-1232.1990 PMC249237

[52] Murata T, Kondo Y, Sugimoto A, Kawashima D, Saito S, Isomura H, et al Epigenetic histone modification of Epstein-Barr virus BZLF1 promoter during latency and reactivation in Raji cells. J Virol. 2012;86(9):4752–61. Epub 2012/02/24. 10.1128/JVI.06768-11 22357272 PMC3347330

[53] Bailey SG, Verrall E, Schelcher C, Rhie A, Doherty AJ, Sinclair AJ. Functional interaction between Epstein-Barr virus replication protein Zta and host DNA damage response protein 53BP1. Journal of Virology. 2009;83(21):11116–22. 10.1128/JVI.00512-09 19656881 PMC2772799

[54] Sun Y, Jiang X, Chen S, Fernandes N, Price BD. A role for the Tip60 histone acetyltransferase in the acetylation and activation of ATM. Proceedings of the National Academy of Sciences of the United States of America. 2005;102(37):13182–7. 16141325 10.1073/pnas.0504211102 PMC1197271

[55] Soutoglou E, Misteli T. Activation of the cellular DNA damage response in the absence of DNA lesions. Science. 2008;320(5882):1507–10. 10.1126/science.1159051 18483401 PMC2575099

[56] Bhende PM, Seaman WT, Delecluse HJ, Kenney SC. The EBV lytic switch protein, Z, preferentially binds to and activates the methylated viral genome. Nature Genetics. 2004;36(10):1099–104. 15361873 10.1038/ng1424

[57] Kalla M, Schmeinck A, Bergbauer M, Pich D, Hammerschmidt W. AP-1 homolog BZLF1 of Epstein-Barr virus has two essential functions dependent on the epigenetic state of the viral genome. Proceedings of the National Academy of Sciences of the United States of America. 2010;107(2):850–5. 10.1073/pnas.0911948107 20080764 PMC2818922

[58] Lehnertz B, Ueda Y, Derijck AAHA, Braunschweig U, Perez-Burgos L, Kubicek S, et al Suv39h-mediated histone H3 lysine 9 methylation directs DNA methylation to major satellite repeats at pericentric heterochromatin. Current Biology. 2003;13(14):1192–200. 12867029 10.1016/s0960-9822(03)00432-9

[59] Vire E, Brenner C, Deplus R, Blanchon L, Fraga M, Didelot C, et al The Polycomb group protein EZH2 directly controls DNA methylation. Nature. 2006;439(7078):871–4. Epub 2005/12/17. 16357870 10.1038/nature04431

[60] Ayoub N, Jeyasekharan AD, Venkitaraman AR. Mobilization and recruitment of HP1: a bimodal response to DNA breakage. Cell cycle (Georgetown, Tex). 2009;8(18):2945–50. 19657222

[61] Soria G, Polo SE, Almouzni G. Prime, Repair, Restore: The Active Role of Chromatin in the DNA Damage Response. Molecular Cell. 2012;46(6):722–34. 10.1016/j.molcel.2012.06.002 22749398

[62] Sun Y, Jiang X, Xu Y, Ayrapetov MK, Moreau LA, Whetstine JR, et al Histone H3 methylation links DNA damage detection to activation of the tumour suppressor Tip60. Nature Cell Biology. 2009;11(11):1376–82. 10.1038/ncb1982 19783983 PMC2783526

[63] Graham FL, van der Eb AJ. A new technique for the assay of infectivity of human adenovirus 5 DNA. Virology. 1973;52(2):456–67. Epub 1973/04/01. 4705382 10.1016/0042-6822(73)90341-3

[64] Delecluse HJ, Hilsendegen T, Pich D, Zeidler R, Hammerschmidt W. Propagation and recovery of intact, infectious Epstein-Barr virus from prokaryotic to human cells. Proceedings of the National Academy of Sciences of the United States of America. 1998;95(14):8245–50. 9653172 10.1073/pnas.95.14.8245 PMC20961

[65] Feederle R, Mehl-Lautscham AM, Bannert H, Delecluse HJ. The Epstein-Barr virus protein kinase BGLF4 and the exonuclease BGLF5 have opposite effects on the regulation of viral protein production. Journal of Virology. 2009;83(21):10877–91. 10.1128/JVI.00525-09 19710145 PMC2772808

[66] Heston L, Rabson M, Brown N, Miller G. New Epstein-Barr virus variants from cellular subclones of P3J-HR-1 Burkitt lymphoma. Nature. 1982;295(5845):160–3. 6276755 10.1038/295160a0

[67] Epstein MA, Achong BG, Barr YM, Zajac B, Henle G, Henle W. Morphological and virological investigations on cultured Burkitt tumor lymphoblasts (strain Raji). Journal of the National Cancer Institute. 1966;37(4):547–59. 4288580

[68] Chang PJ, Shedd D, Gradoville L, Cho MS, Chen LW, Chang J, et al Open reading frame 50 protein of Kaposi's sarcoma-associated herpesvirus directly activates the viral PAN and K12 genes by binding to related response elements. Journal of Virology. 2002;76(7):3168–78. 11884541 10.1128/JVI.76.7.3168-3178.2002 PMC136055

[69] Abate C, Patel L, Rauscher FJ 3rd, Curran T. Redox regulation of fos and jun DNA-binding activity in vitro. Science. 1990;249(4973):1157–61. Epub 1990/09/07. 2118682 10.1126/science.2118682

[70] Francis AL, Gradoville L, Miller G. Alteration of a single serine in the basic domain of the Epstein-Barr virus ZEBRA protein separates its functions of transcriptional activation and disruption of latency. Journal of Virology. 1997;71(4):3054–61. 9060666 10.1128/jvi.71.4.3054-3061.1997 PMC191435

[71] Chen MR, Chang SJ, Huang H, Chen JY. A protein kinase activity associated with Epstein-Barr virus BGLF4 phosphorylates the viral early antigen EA-D in vitro. Journal of Virology. 2000;74(7):3093–104. 10708424 10.1128/jvi.74.7.3093-3104.2000 PMC111808

[72] El-Guindy AS, Miller G. Phosphorylation of Epstein-Barr virus ZEBRA protein at its casein kinase 2 sites mediates its ability to repress activation of a viral lytic cycle late gene by Rta. Journal of Virology. 2004;78(14):7634–44. 15220438 10.1128/JVI.78.14.7634-7644.2004 PMC434091

[73] Pearson GR, Vroman B, Chase B, Sculley T, Hummel M, Kieff E. Identification of polypeptide components of the Epstein-Barr virus early antigen complex with monoclonal antibodies. J Virol. 1983;47(1):193–201. Epub 1983/07/01. 6306272 10.1128/jvi.47.1.193-201.1983 PMC255226

[74] El-Guindy A, Ghiassi-Nejad M, Golden S, Delecluse HJ, Miller G. Essential role of Rta in lytic DNA replication of Epstein-Barr virus. Journal of Virology. 2013;87(1):208–23. 10.1128/JVI.01995-12 23077295 PMC3536415

[75] Philpott NJ, Turner AJ, Scopes J, Westby M, Marsh JC, Gordon-Smith EC, et al. The use of 7-amino actinomycin D in identifying apoptosis: simplicity of use and broad spectrum of application compared with other techniques. Blood. 1996(0006–4971 (Print)). 8630384

[76] Daigle D, Gradoville L, Tuck D, Schulz V, Wang'ondu R, Ye J, et al Valproic Acid Antagonizes the Capacity of Other Histone Deacetylase Inhibitors To Activate the Epstein-Barr Virus Lytic Cycle. Journal of Virology. 2011;85(11):5628–43. 10.1128/JVI.02659-10 21411522 PMC3094991

[77] El-Guindy A, Heston L, Delecluse HJ, Miller G. Phosphoacceptor site S173 in the regulatory domain of Epstein-Barr virus ZEBRA protein is required for lytic DNA replication but not for activation of viral early genes. Journal of Virology. 2007;81(7):3303–16. 17215287 10.1128/JVI.02445-06 PMC1866087

[78] Tokuyasu KT. Visualization of longitudinally-oriented intermediate filaments in frozen sections of chicken cardiac muscle by a new staining method. J Cell Biol. 1983;97(2):562–5. Epub 1983/08/01. 6193126 10.1083/jcb.97.2.562 PMC2112529

