# Supplementary figures and images for: DNA Damage Signaling Is Induced in the Absence of Epstein—Barr Virus (EBV) Lytic DNA Replication and in Response to Expression of ZEBRA
Source: PLoS One. 2015 May 7;10(5):e0126088. doi: 10.1371/journal.pone.0126088 (PMC4423948; doi:10.1371/journal.pone.0126088)

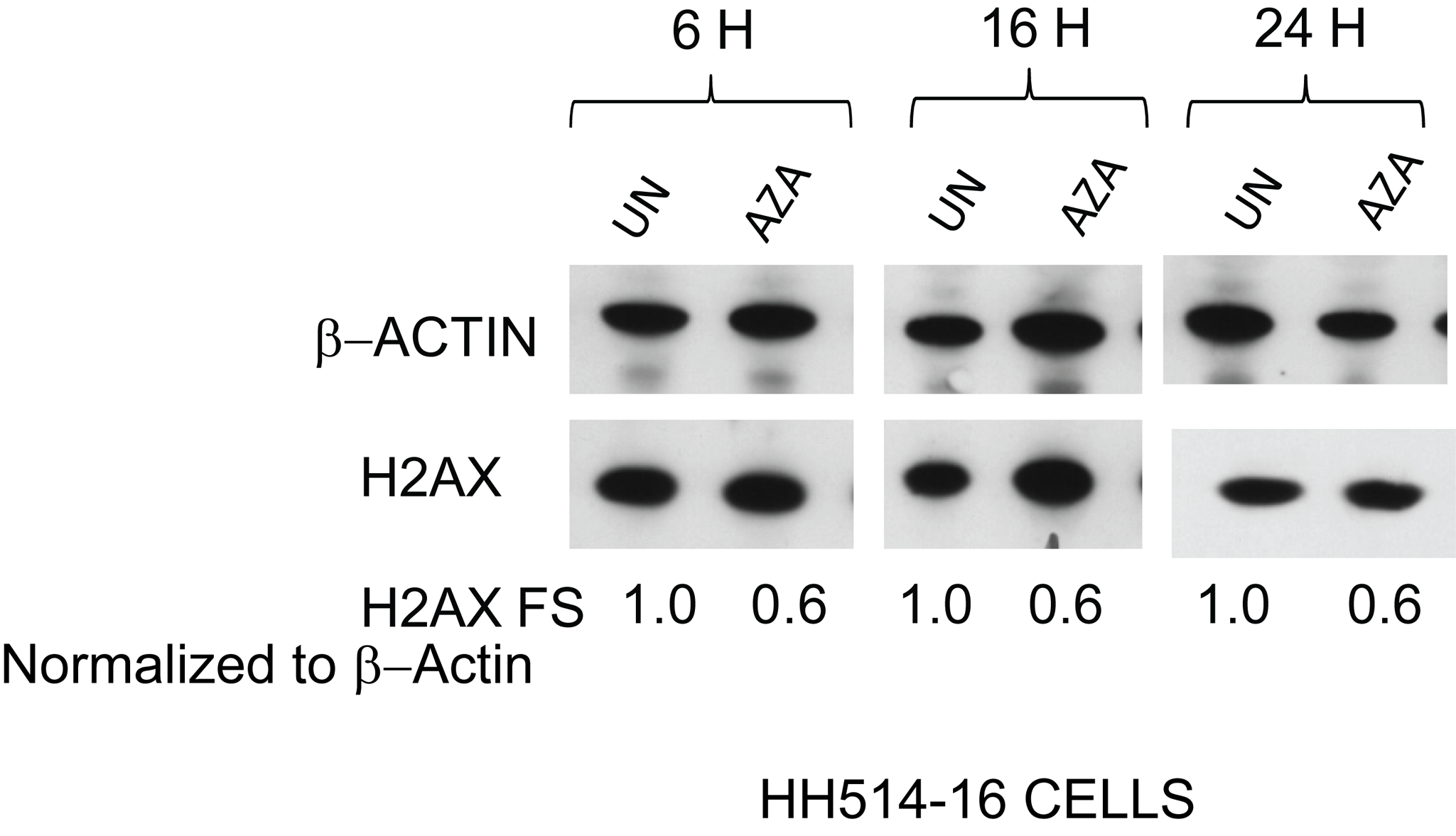

Supplement: S1 Fig — Cell lysates from HH514-16 cells not treated (UN) or treated with 5-aza-2’-deoxycytidine (AZA) for 6, 16, or 24 hours, used in Fig 4E, were analyzed by immunoblots with antibodies against β-actin and H2AX (S135). The indicated average fold-induction values of H2AX, based on densitometry values of H2AX bands normalized to unphosphorylated β-actin bands in untreated versus AZA samples at each time point, were used in calculations of fold induction values of γH2AX bands normalized to unphosphorylated H2AX in Fig 4F. (TIF) [file pone.0126088.s001.tif]

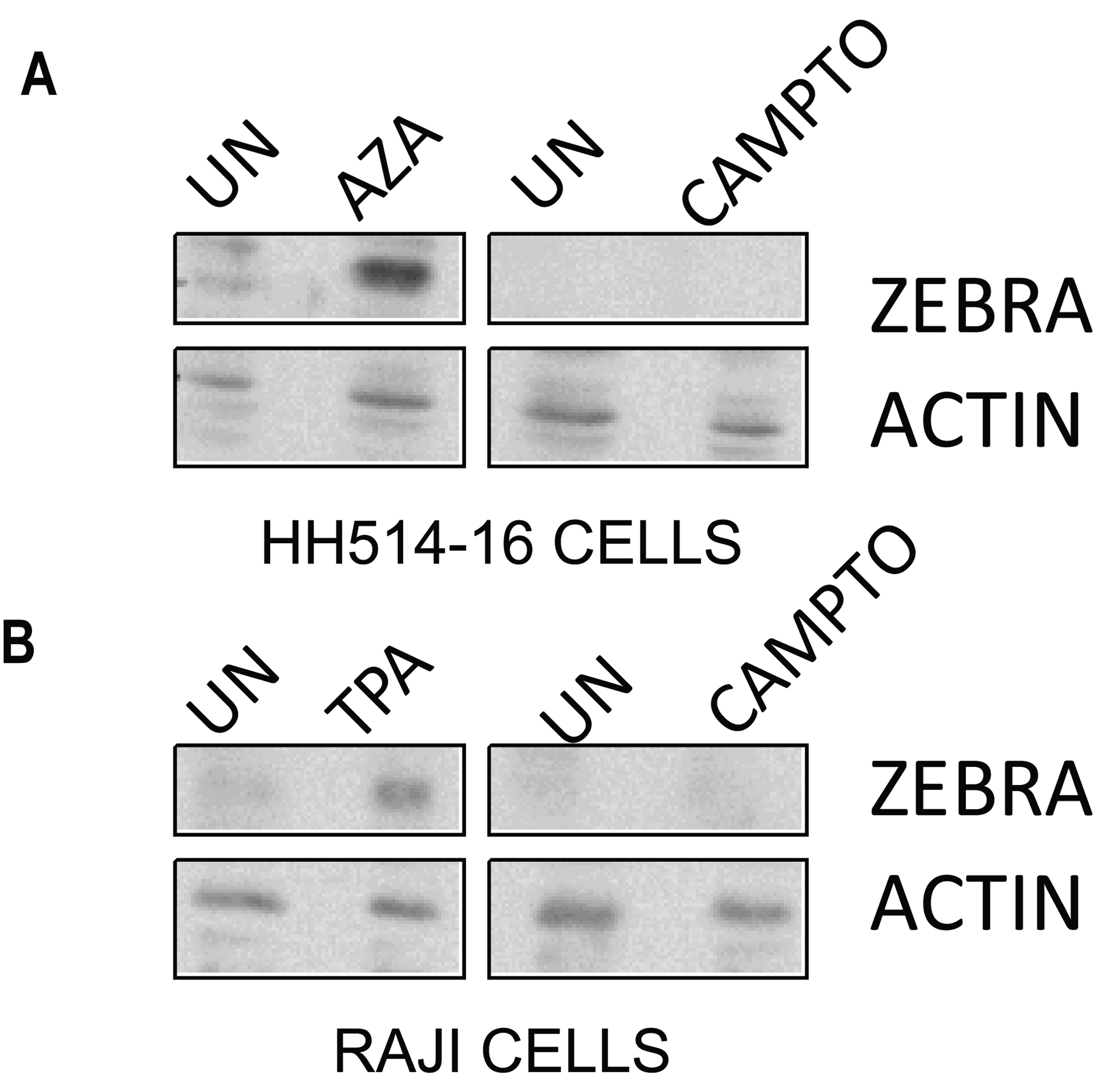

Supplement: S2 Fig — Cell lysates from (A) HH514-16 cells untreated or treated with AZA for 24 hours or camptothecin (CAMPTO) or (B) Raji cells untreated or treated with TPA or Camptothecin were analyzed by immunoblots with antibodies against ZEBRA and β-actin. In both panels, camptothecin was washed off after 2 hours of treatment and cells incubated for an additional 22 hours. (TIF) [file pone.0126088.s002.tif]

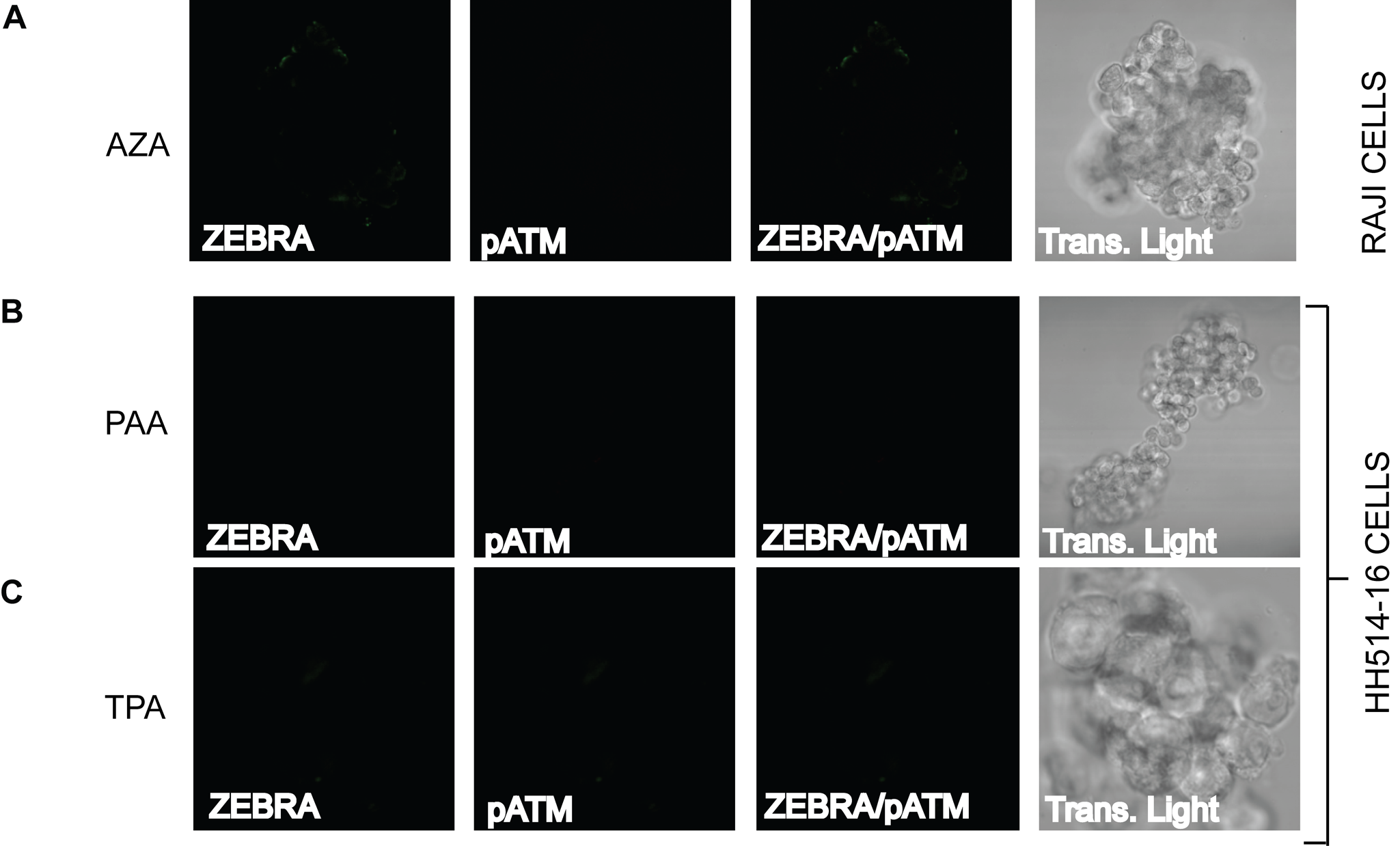

Supplement: S3 Fig — (A) Raji cells treated with AZA or HH514-16 cells treated with (B) PAA or (C) TPA were double-stained for ZEBRA and pATM (S1981). (TIF) [file pone.0126088.s003.tif]

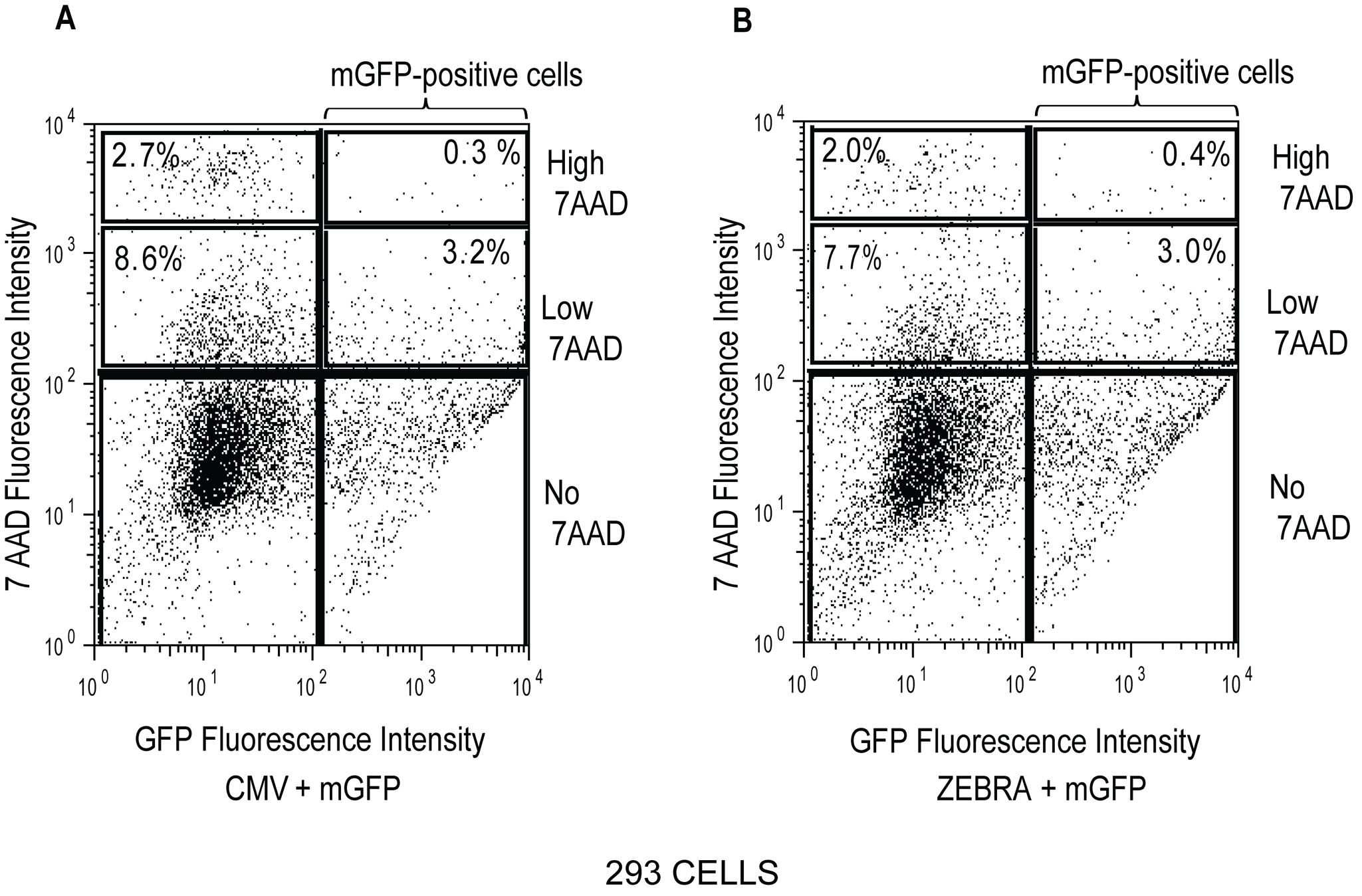

Supplement: S4 Fig — 293 cells were transfected with CMV (B, i) or WTZ (B, ii) and a plasmid bearing prenylated GFP that localizes to the membrane (mGFP), as a marker for transfected cells. After 32 hours, cells were treated with 7-Amino-actinomycin D (7AAD) and analyzed by flow cytometry to detect apoptotic (low 7AAD staining) and dead cells (high 7AAD staining). Flourescent activated cell sorting (FACS) plots of cells are shown. The percentages of total GFP positive or negative cells with high, low or no 7AAD staining are shown. (TIF) [file pone.0126088.s004.tif]

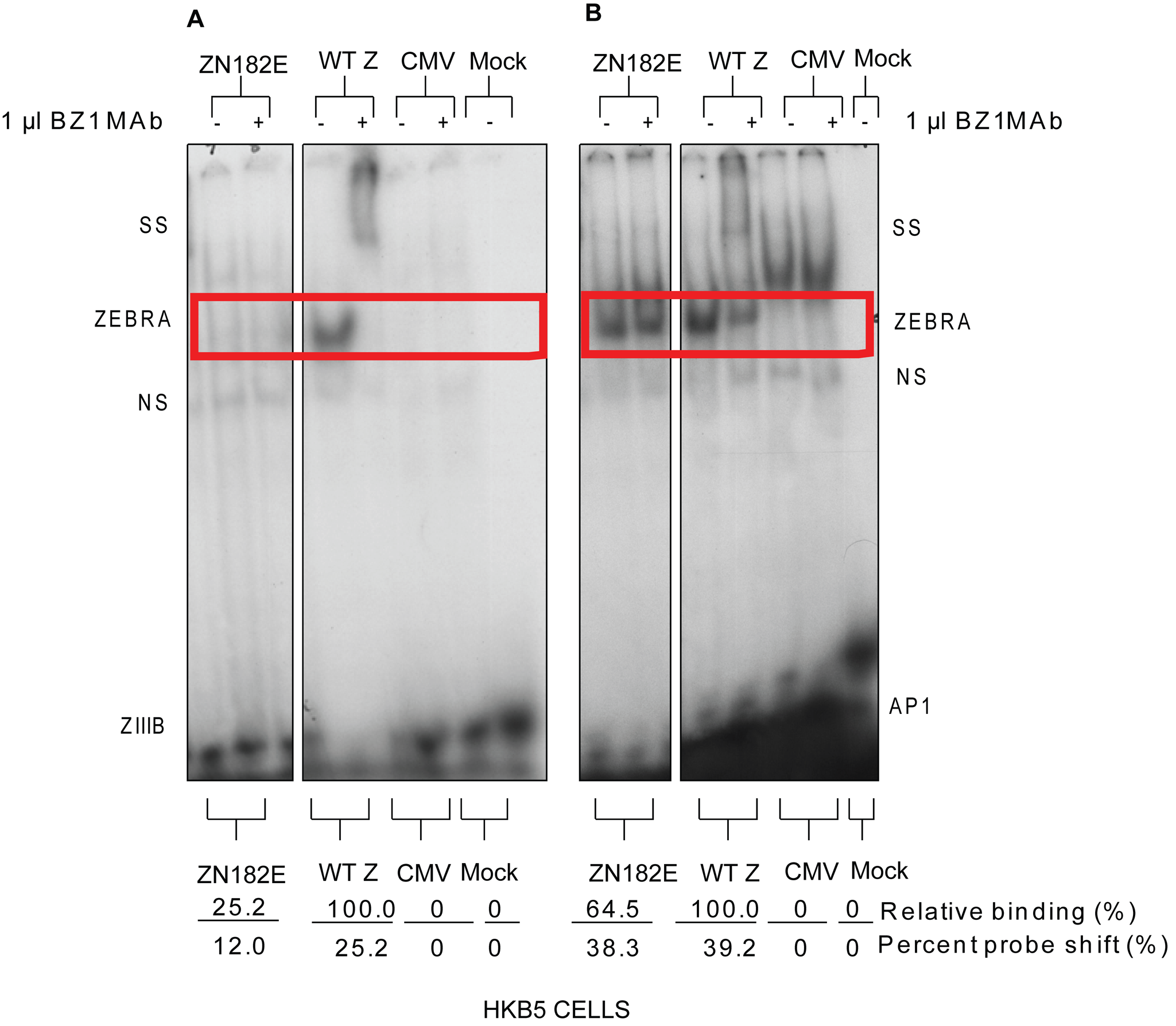

Supplement: S5 Fig — HKB5/B5 cells were transfected with Z(N182E), wild type ZEBRA (WTZ), or CMV plasmids, or mock-transfected (Mock). Shown are EMSAs using whole cell extracts of transfected cells. Relative binding of Z(N182E) or WT Z to a radioactive probe containing A) a ZIIIB DNA sequence (TTAGCAA) or B) AP1 DNA sequence (TGAGTCA) was determined using 1 μl of a monoclonal antibody to ZEBRA (BZ1 MAb). SS denotes super shifted band; NS denotes non-specific band. (TIF) [file pone.0126088.s005.tif]

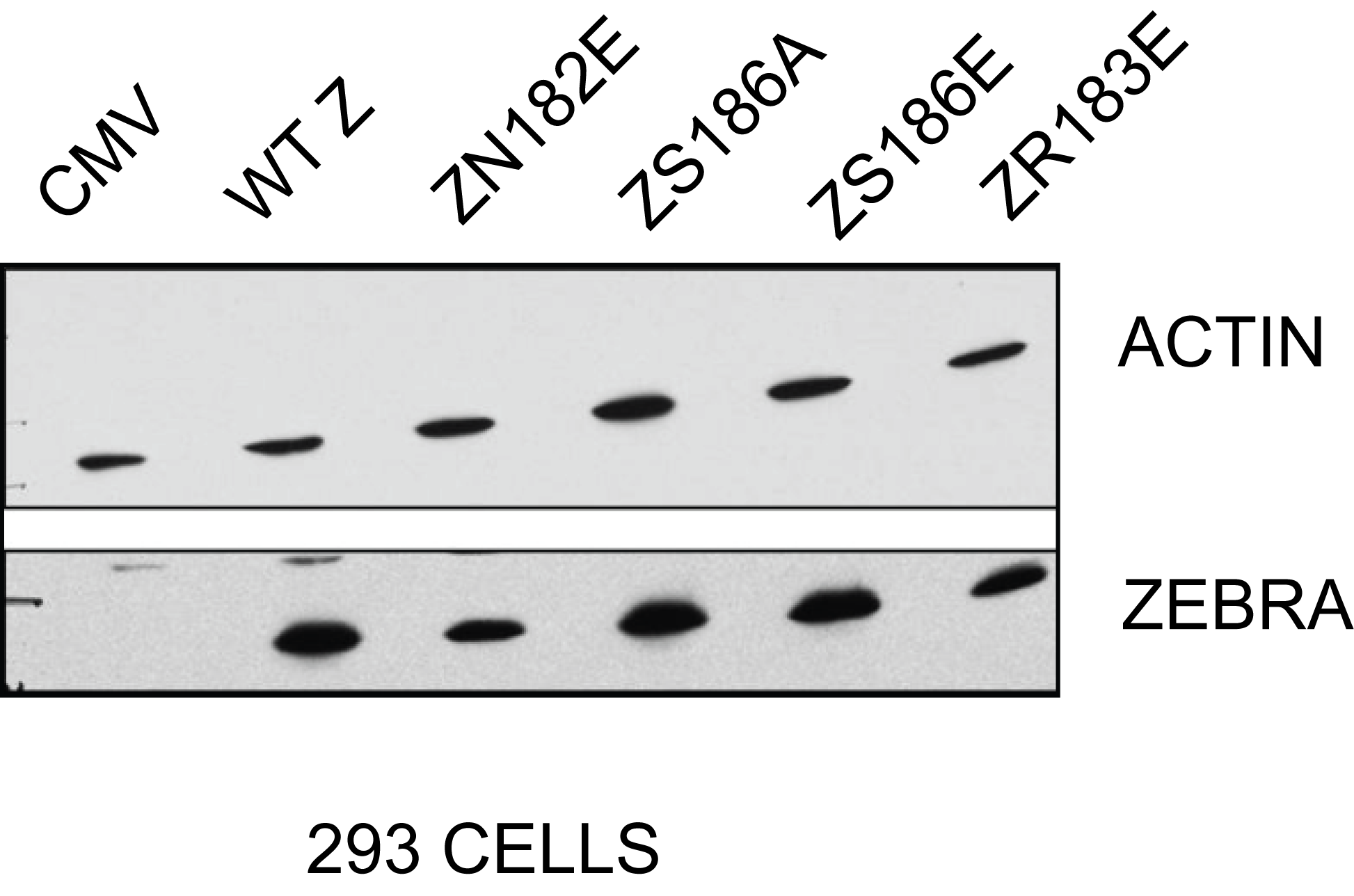

Supplement: S6 Fig — Cell lysates of 293 cells transfected with wild type ZEBRA, ZN182E, ZS186A, ZS186E, or ZR183E mutants were analyzed by immunoblots with antibodies against ZEBRA and β-ACTIN. (TIF) [file pone.0126088.s006.tif]
